# Behavioral and Molecular Characterization of Prenatal Stress Effects on the C57BL/6J Genetic Background for the Study of Autism Spectrum Disorder

**DOI:** 10.1523/ENEURO.0186-23.2024

**Published:** 2024-02-09

**Authors:** Jeffrey T. Dunn, Alessandro Guidotti, Dennis R. Grayson

**Affiliations:** ^1^Department of Psychiatry, University of Illinois Chicago, Chicago, Illinois 60612; ^2^Department of Psychology, University of Illinois Chicago, Chicago, Illinois 60607; ^3^Department of Psychiatry and Behavioral Sciences, Northwestern University, Chicago, Illinois 60611

**Keywords:** behavior, C57BL/6, epigenetics, gene expression, gestational stress

## Abstract

Stress-inducing events during pregnancy are associated with aberrant neurodevelopment resulting in adverse psychiatric outcomes, including autism spectrum disorder (ASD). While numerous preclinical models for the study of ASD are frequently generated using C57BL/6J mice, few studies have investigated the effects of prenatal stress on this genetic background. In the current manuscript, we stressed C57BL/6 dams during gestation and examined numerous behavioral and molecular endophenotypes in the adult male and female offspring to characterize the resultant phenotype as compared with offspring born from nonstressed (NS) dams. Adult mice born from prenatal restraint stressed (PRS) dams demonstrated reduced sociability and reciprocal social interaction along with increased marble burying behaviors relative to mice born from nonstressed control dams. Differential expression of genes related to excitatory and inhibitory neurotransmission was evaluated in the medial prefrontal cortex, amygdala, hippocampus, nucleus accumbens and caudate putamen via qRT-PCR. The male PRS mouse behavioral phenotype coincided with aberrant expression of glutamate and GABA marker genes (e.g., Grin1, Grin2b, Gls, Gat1, Reln) in neural substrates of social behavior. Rescue of the male PRS sociability deficit by a known antipsychotic with epigenetic properties (i.e., clozapine (5 mg/kg) + 18 hr washout) indicated possible epigenetic regulation of genes that govern sociability. Clozapine treatment increased the expression levels of genes involved in DNA methylation, histone methylation, and histone acetylation in the nucleus accumbens. Identification of etiology-specific mechanisms underlying clinically relevant behavioral phenotypes may ultimately provide novel therapeutic interventions for the treatment of psychiatric disorders including ASD.

## Significance Statement

The effects of prenatal stress on the C57BL/6J mouse genetic background are incompletely understood with regard to adverse psychiatric outcomes and respective underlying molecular mechanisms. Considering the prominent use of C57BL/6J mice for the generation of preclinical models for the study of autism spectrum disorder (ASD) and putative differences in stress resiliency across mouse strains, this gap in knowledge is an obstacle to contextualizing prenatal stress as an ASD etiological risk factor within the broader ASD preclinical literature. This study expands current knowledge of the relationship between prenatal stress exposure and the subsequent presentation of representative behaviors for ASD symptom domains, underlying changes in gene expression, and insight into the potential of alleviating ASD-like behaviors through epigenetic mechanisms.

## Introduction

Although the effects of prenatal restraint stress (PRS) have previously been characterized in rats (Van den Hove et al., 2005, 2014; Zuena et al., 2008; Yeh et al., 2012, Sun et al., 2013) and strains of mice such as the SWA-ND4 mouse (Matrisciano et al., 2013; Dong et al., 2015), less is known about the effects of prenatal stress in C57BL/6J mice, which are commonly used for the generation of genetic mouse models of various disorders, including autism spectrum disorder (ASD); C57BL/6J mice are additionally used as controls for mouse models of idiopathic ASD such as the BTBR mouse (Moy et al., 2004; Amodeo et al., 2012). Behaviorally and molecularly phenotyping a model of PRS on the same genetic background as that most commonly used in the generation of other mouse models for the study of ASD allows the scientific community to better contextualize the effects of PRS relative to other proposed ASD etiological risk factors. ASD is a neurodevelopmental disorder characterized by social communication and interaction deficits and the presence of restricted, repetitive patterns of behavior that persist throughout the lifespan. Current knowledge of the C57BL/6J PRS behavioral phenotype is limited to the domains of anxiety, learned helplessness, sociability, and spatial memory (Zhao et al., 2013; Akatsu et al., 2015; Benoit et al., 2015; Gur et al., 2019).

The current investigation has expanded and strengthened the presently underdeveloped understanding of the C57BL/6J PRS mouse behavioral phenotype as it pertains to ASD by examining behaviors relevant to both the social and restrictive, repetitive patterns of behavior (RRB) symptom domains of the clinical condition. Investigation of social behavior in PRS mice was conducted through the implementation of the three-chamber test of sociability, three-chamber social recognition test, and a reciprocal social interaction assay. The extent to which PRS mice exhibit RRBs was investigated using a marble-burying task and assessment of self-grooming behavior. Comorbid anxiety disorders have been identified in 40% of ASD cases (Zaboski and Storch, 2018) and are associated with higher rates of RRB expression (Sukhodolsky et al., 2008; Joosten et al., 2009; Rodgers et al., 2012). Anxiety-like behaviors were therefore measured in PRS mice using the elevated plus maze (EPM) and light/dark box behavioral assays to allow for the investigation of a relationship between anxiety and the hypothesized PRS-induced RRBs. In addition, the possibility exists that differences in the expression of RRBs might be explained by underlying differences in general motor activity between NS and PRS mice. We determined whether any differences exist in locomotor activity by measuring both horizontal and vertical locomotor activity in an open field. Together, the present battery of behavioral assays provides a thorough characterization of C57BL/6J PRS mouse behaviors across ASD symptom domains that is insufficiently represented in the current literature.

Consideration of PRS mice as a preclinical model for the study of ASD prompted the pursuit of the neural bases of PRS-induced social deficits and/or RRBs through the lens of the prominent ASD hypothesis of excitation-inhibition (E/I) imbalance within behaviorally-relevant neural circuits. At the molecular level, signs of E/I dysregulation may be apparent in the form of atypical expression of genes encoding protein products involved in the glutamatergic and GABAergic neurotransmitter systems. Therefore, the present experiments measure the relative expression of C57BL/6J PRS glutamatergic (i.e., Grin1, Grin2b, Vglut1, Vglut2, Gls, Gls2, Glul) and GABAergic (i.e., Gad1, Gad2, Reln, Pvalb, Sst, Gat1, Gat3) gene markers in brain regions implicated in ASD social deficits (i.e., mPFC, Amg, Hpc, NAc) and RRBs (i.e., mPFC, CPu, NAc) through quantitative real-time polymerase chain reaction (qRT-PCR) experiments. Presently, the only report of aberrant gene expression in C57BL/6J PRS mice that implicates an E/I imbalance is a reported decrease in the expression of hippocampal Grin2B mRNA (Zhao et al., 2013). In that light, the present experiments function to provide the field with an initial framework for conceptualizing molecular contributions to potential E/I imbalances in C57BL/6J PRS mice, as it pertains to the study of ASD-relevant behaviors.

## Materials and Methods

### Animals and stress procedure

Timed-pregnant female C57BL/6J mice were ordered from the Jackson Laboratory to arrive on gestational day E6/7. Pregnant mice were individually housed (28 cm wide × 17 cm long × 12 cm high plastic cage) with corn cob bedding and two paper tubes which were available for use as nesting material and environmental enrichment. Pregnant dams were pseudorandomly assigned to a prenatal stress condition on day E8/E9 based on weight to maximize the likelihood of a similar number of pups being born to each group. Dams belonging to the nonstressed (NS) condition were left undisturbed in their respective home cages throughout the gestational period. Dams belonging to the PRS condition were placed in a clear plastic tube (12 cm × 3 cm) under a bright light for 45 min three times per day from E8/9 through delivery (Dong et al., 2015).

Following birth, all mice were returned to their respective dams and allowed to nurse. Offspring of dams assigned to the NS condition are referred to as NS mice and offspring of dams assigned to the PRS condition are referred to as PRS mice. Pups were weaned from their mother at 21 d of age, at which time they were group housed with same sex littermates until the conclusion of the experiments. All behavioral assays were conducted using adult (i.e., 10–14 weeks of age) male and female C57BL/6J NS and PRS mice. A total of 216 mice were tested in this study (Male: NS = 52, PRS = 56; Female: NS = 58, PRS = 50) with a minimum of three litters represented in each experimental group for all assays.

All mice were housed in a temperature and humidity-controlled room with a 12 h light/dark cycle (lights on at 6:00 A.M.) and received food and water ad libitum throughout the experiments. All animal care and use were in accordance with the National Institutes of Health Guide for the Care and Use of Laboratory Animals and approved by the Institutional Laboratory Animal Care and Use Committee at the University of Illinois Chicago.

### Drug preparation and administration

A clozapine (CLZ) (Sandoz Pharmaceuticals) solution was prepared at a dose of 5 mg/kg body weight. CLZ was dissolved in sterile water and glacial acetic acid, and pH balanced to pH = 7 with dropwise additions of 30% NaOH 10N. Intraperitoneal injections of clozapine were administered two times per day for 5 d. An 18 h washout period was implemented between the final CLZ injection and behavioral testing.

### Behavioral testing

Mice were allowed to habituate to the behavioral testing room for 1 h prior to the initiation of any behavioral assay. All behavioral testing occurred between 8:00 A.M. and 3:00 P.M. to mitigate potential influence of the murine diurnal corticosterone rhythm. Two days of rest were instituted between behavioral tests, with the order of testing counterbalanced across cohorts. Order effects were mitigated through the implementation of three testing sequences: testing sequence 1: EPM, locomotor activity, marble burying, reciprocal social interaction; testing sequence 2: self-grooming, EPM, three-chamber social interaction, locomotor activity; and testing sequence 3: locomotor activity, three-chamber social interaction, self-grooming, light/dark box. Within the three-chamber social interaction test, the lateral chamber containing the social stimulus (i.e., novel mouse) and the particular mouse used as the social stimulus were both counterbalanced.

Individual mice did not undergo testing on multiple qualitatively similar tests (e.g., measures of anxiety-like behavior). Testing apparatuses were cleaned with 70% Ethanol before and after use by each mouse.

#### Self-grooming

Testing of repetitive self-grooming behavior was conducted using an empty, clear, plastic cage (28 cm wide × 17 cm long × 12 cm high) covered with a clear, plastic cage filter top. Once in the testing container, mice were allowed to freely explore for a period of 20 min. The first 10 min served as a habituation period. During the final 10 min of testing, a trained observer recorded the cumulative amount of time spent grooming all body regions. Grooming was defined in accordance with the four phases described by Berridge et al. (2005). In brief, self-grooming was defined as licking of the head region (e.g., snout, over one or both ears) via forepaws, as well as licking of the torso or tail. The cumulative duration of time spent grooming was calculated from engagement in these individual grooming behaviors.

#### Marble burying

The marble burying assay was conducted in a clear plastic container (28 cm wide × 17 cm long × 12 cm high) filled with 3 cm of corn cob bedding and covered with a clear plastic filter top. To assess marble burying behavior, 20 glass marbles (1.5 cm diameter) were placed on top of the bedding material in a 4 × 5 array and mice were allowed to freely explore the arena and marbles for 30 min. Mice were removed from the testing arena at the conclusion of the 30 min testing period and all marbles that were ≥2/3 covered by bedding material were considered as buried. A count of buried marbles was taken as measurement for analysis of marble burying behavior.

#### Locomotor activity

Locomotor activity was assessed in a perspex box (20 cm long × 20 cm wide × 20 cm high) equipped with horizontal and vertical infrared sensor beams around the interior perimeter of the arena. Connection of the perspex box to a computerized animal activity monitoring system running VersaMax software enabled the tracking of horizontal and vertical locomotor activity through disruptions of the horizontal and vertical sensor beams, respectively. Mice were placed in the center of the box and allowed to freely navigate the open field for 10 min.

#### Elevated-plus maze (EPM)

A Hamilton Kinder EPM connected to a Windows-compatible computer running MotorMonitor software was used to detect and record the cumulative amount of time spent in the open arms (5 cm wide × 38 cm long), the closed arms (5 cm wide × 38 cm long; walls 15 cm high), and the intersection area (5 cm wide × 5 cm long) of the EPM apparatus (77.5 cm high). Testing was conducted under dim light with a dark curtain enclosure surrounding the EPM. Mice were placed in the center (i.e., intersection) of the EPM, oriented toward a closed arm, and were allowed to freely explore the apparatus for 5 min.

#### Light/dark box

Exploration of contrasting light/dark environments created by equally sized black acrylic (i.e., “dark”) and clear acrylic (i.e., “light”) chambers (40 cm wide × 20 cm long × 35 cm high) conjoined by an opening (10 cm wide × 5 cm high) in a black acrylic partition was video recorded and scored by a trained observed using the JWatcher software. Ambient light (0.25 Amp; light-emitting diode light) perceptible in the light chamber was blocked from the dark chamber by placing a black acrylic lid on top of the black acrylic walls and partition. Mice were placed in the dark chamber and allowed to freely navigate the arena for 5 min. Cumulative duration of time spent in each chamber was measured for analysis of anxiety-like behavior.

#### Three-chamber social interaction – sociability

Sociability was assessed in a rectangular, clear plexiglass arena containing three equally sized chambers (20 cm long × 40.5 cm wide × 22 cm high each) connected by a 10 cm wide × 5 cm high opening in the center of each of the two plexiglass walls that delineate the center chamber from the lateral chambers of the apparatus. A novel same-sex conspecific placed under an inverted wire cup served as the social stimulus and an empty inverted wire cup served as the nonsocial stimulus. Inverted wire cups were centrally placed within lateral chambers for stimuli presentation. Mice underwent a 5 min habituation period during which they were able to freely explore the arena. Upon completion of the arena habituation period, mice were guided to the center chamber and doors to the lateral chambers were lowered in preparation for sociability testing. The social stimulus and nonsocial stimulus were then placed within opposing lateral chambers. Sociability testing began with the removal of the lateral chamber doors, and the mouse was permitted to freely explore the three chambers for 10 min. The cumulative duration of sniffing behavior directed toward each stimulus was measured from video recordings of the three-chamber sociability assay using JWatcher software. Sniffing behavior was defined as the snout being oriented toward a stimulus at a distance of ≤1 cm from that same stimulus. A sociability index score was calculated for each mouse as {[social sniffing/(social sniffing + nonsocial sniffing)] × 100}.

#### Reciprocal social interaction

Testing of reciprocal social interaction was adapted from the protocol of Chang et al. (2017). Mice were placed with a novel same-sex conspecific in a clean, clear plastic cage (28 cm wide × 17 cm long × 12 cm high) with clean corn cob bedding and a clear plastic filter top for a period of 30 min. Frequency and duration of social (i.e., nose-nose/nose-anogenital/nose-side sniffing, following, push-crawl behavior) and nonsocial (i.e., self-grooming, arena exploration) behaviors were measured from video recordings of the behavioral testing session using JWatcher software on a VLC media player-equipped computer.

### Molecular assays

#### Tissue collection

Upon completion of behavioral experiments, mice were sacrificed by cervical dislocation for the collection of brain tissue. NS and PRS mPFC (+0.86 to +2.86 from the bregma), Amg (−1.06 to −2.06 from the bregma), Hpc (−2.06 to −4.06 from the bregma), CPu ((+0.86 to +2.86 from the bregma), and NAc ((+0.86 to +2.86 from the bregma) were dissected from fresh tissue slices over dry ice and stored at −80°C until use. Samples classified as mPFC are defined here as a homogenate of ACC, PL, and IL.

#### Quantitative real-time polymerase chain reaction experiments

Fresh frozen NS (*n* = 7) and PRS (*n* = 5) mouse mPFC, Hpc, Amg, NAc, and CPu tissue samples were individually weighed and placed into BeadBug tubes prefilled with 1.5 mm zirconium beads and 700 µl TRIzol reagent for total RNA isolation by disruption and homogenization using a BeadBug microtube homogenizer at 3,500 rpm for 60 s. RNA samples were further purified with the RNeasy micro kit (Qiagen). A Qubit 3.0 fluorometer (Invitrogen/Life Technolgies) was used to determine the concentration of RNA in each extracted sample. All samples were brought to an equal concentration of RNA via dilution with RNAse free water prior to reverse transcription. Complementary DNA (cDNA) was synthesized in a GeneAmp PCR System 9,700 (PE Applied Biosystems) from the diluted RNA using a M_MLV reverse transcriptase master mix (Invitrogen) under the following conditions: ramp up to 25°C and plateau for 10 min, 37°C for 60 min, repeat 37°C for 60 min, and 70°C for 15 min, ramp down to 4°C. qRT-PCR experiments were conducted using the synthesized cDNA and a SYBR green master mix in a Stratagene Mx3005P thermocycler (Agilent Technologies) under the following conditions: (segment 1) ramp up to 95°C and plateau for 10 min, (segment 2) 40 cycles of 95°C for 30 s, ramp down to 60°C and maintain for 1 min, (segment 3) ramp up to 95°C and plateau for 1 min, ramp down to 55°C and maintain for 30 s, ramp up to 95°C and plateau for 30 s. All samples were tested in duplicate and the reference gene *Hprt1* was used as an external control for normalizing mRNA expression values (i.e., Ct values) of all genes-of-interest (GoI). Changes in gene expression were calculated using the delta delta Ct method. The expression of gene markers of the glutamatergic (i.e., *Grin1, Grin2b, Vglut1, Vglut2, Gls, Gls2, Glul*) and GABAergic (i.e., *Gad1, Gad2, Reln, Pvalb, Sst, Gat1, Gat3*) neurotransmitter systems were assessed in each the mPFC, Amg, Hpc, NAc, and CPu.

#### RNA fluorescent barcoding

Multiplex measurement of 98 GoI (Extended Data Table 1-1) was performed using RNA fluorescent barcoding; GoI selection emphasized genes involved in epigenetic mechanisms previously linked to CLZ, with additional inclusions informed by ingenuity pathway analysis findings from RNA-seq results of a parallel PRS mouse study (Grayson, unpublished). A custom CodeSet/ProbeSet (NanoString Technologies) was designed to measure GoI transcript counts from the NAc of male NS (VEH *n* = 7, CLZ *n* = 6) and PRS (VEH *n* = 5, CLZ *n* = 6) mice used in the described CLZ behavioral pharmacology experiments. In addition to GoI, five reference genes (i.e., *Cyc1, Gusb, Hprt, Rplp0, Top1*) were measured. The geometric mean of the transcript counts for each reference gene was used for normalization. Eight negative controls and six positive controls (NanoString Technologies) were measured as a component of quality control (QC).

Hybridization of reporter and capture probes to the RNA samples was conducted in accordance with the manufacturer’s protocol (NanoString Technologies, MAN-10056-04). Briefly, 50 ng of total RNA at a concentration of 10 ng/µl was incubated with a Reporter CodeSet-hybridization buffer (NanoString Technologies, item no. 000136) master mix and Capture ProbeSet in a thermocycler at 65°C for 24 h. Incubation temperature was then reduced to 4°C until sample processing on the following day. Hybridized samples were brought to a volume of 30 µl with RNAse-free water and loaded into an nCounter SPRINT cartridge (NanoString Technologies, item #100078), which was run on an nCounter SPRINT profiler.

All measured samples were included for analysis, as binding density QC indicated sufficient RNA abundance without lane oversaturation, the fields of view imaging QC was passed, and assessment of positive control linearity yielded *r*^2^ = 1.0 for each sample.

### Experimental design and statistical analysis

The experiments carried out in the present study are intended to serve as a characterization of the C57BL/6J PRS mouse model and do not contain hypotheses pertaining to sex differences. Therefore, the data acquired from male and female mice were analyzed separately to avoid the determination of whether an endophenotype exists in each sex being obfuscated by statistical variance clearly attributable to a nonexperimental variable (i.e., the biological variable of sex). In consideration of the more robust behavioral phenotype in male PRS mice than female PRS mice, existing evidence indicating significant relationships between estrous cycle stage, gene expression, and chromatin architecture (Rocks et al., 2022), and the lack of estrous phase data in the present investigation, only male mice were used for subsequent molecular (i.e., qRT-PCR and RNA fluorescent barcoding) and epigenetically oriented behavioral pharmacology assays (i.e., CLZ).

All *t* tests and analyses of variance (ANOVAs) were calculated using GraphPad Prism (v9.0). The significance threshold of *p *< 0.05 was applied to the interpretation of all behavioral and qRT-PCR results. Independent samples *t* tests were used to evaluate differences between NS and PRS mice in the behavioral assays of repetitive self-grooming, marble burying, locomotor activity, and cumulative reciprocal social interaction. Two-way ANOVAs were used to assess differences between NS and PRS mice across levels of an apparatus factor (i.e., stimuli) in the light/dark box, EPM, and three-chamber test of sociability. A repeated measures two-way ANOVA was used to evaluate differences in reciprocal social interaction between NS and PRS mice across time. Significant interaction effects detected by any two-way ANOVA were followed up using post hoc Tukey’s multiple comparisons tests. Individual qRT-PCR experiments comparing gene expression levels of NS and PRS mice were analyzed by independent samples *t* tests. A Welch’s correction was applied to analyses involving groups with unequal variances. Grubb’s test was used for the detection of outliers. A two-way ANOVA (stress treatment × drug treatment) was performed for each gene on the RNA fluorescent barcoding panel using normalized transcript counts calculated by the nSolver Analysis Software (v4.0). In consideration of the multiplex measurement of RNA fluorescent barcoding data, a false discovery rate (FDR) was calculated using Microsoft Excel to account for the chance occurrence of any main effect of stress condition, main effect of drug treatment, or interaction effect in our dataset. Statistical significance of RNA fluorescent barcoding results was determined using a threshold of FDR < 0.05.

## Results

### Investigation of an ASD-relevant behavioral phenotype in male and female C57BL/6J PRS mice

#### Three-chamber test of sociability

**Figure 1.**
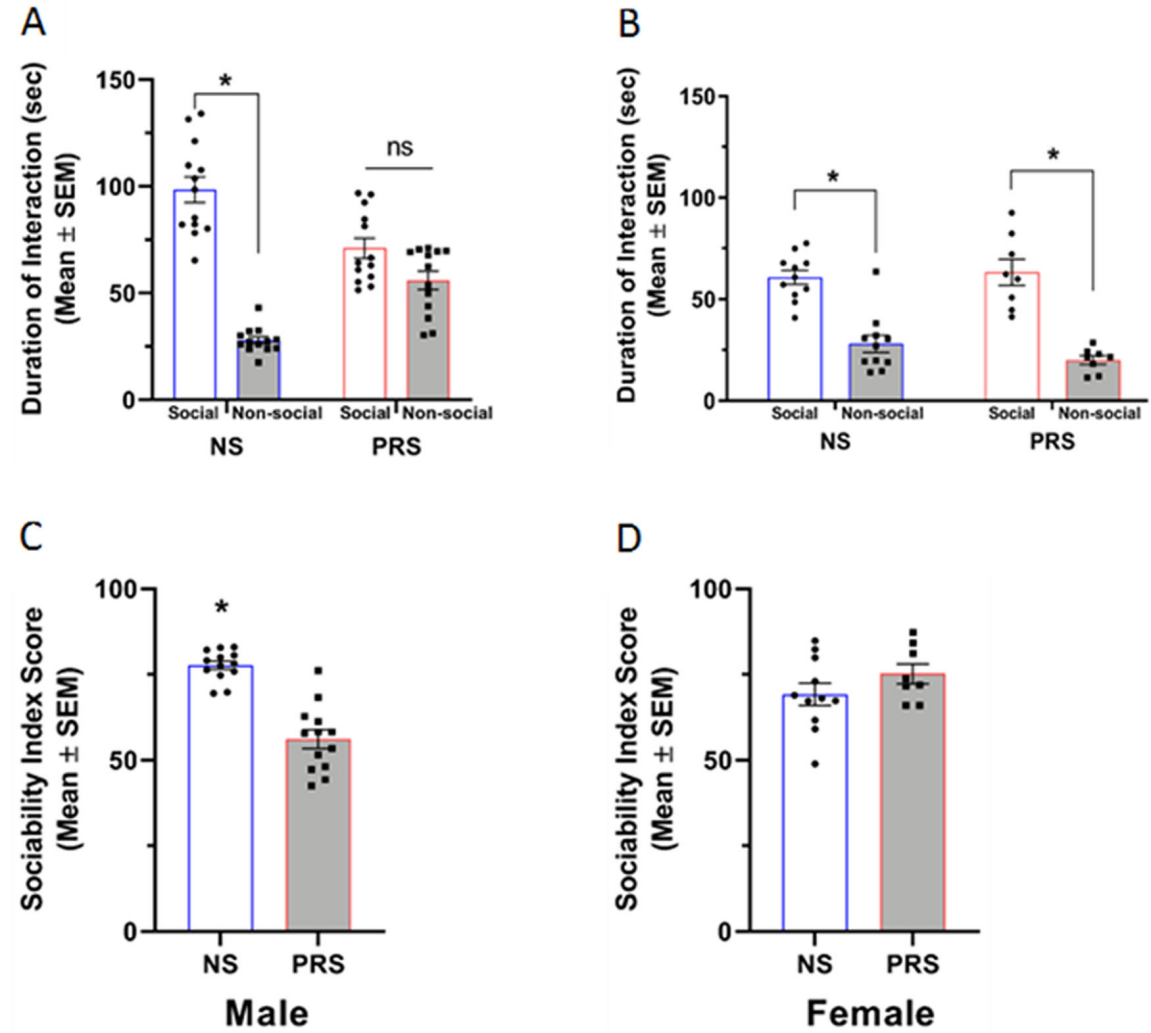
***A***, Male NS (*n* = 13) but not PRS (*n* = 13) mice exhibit typical social behavior by showing a significant preference for interacting with a social stimulus over a nonsocial stimulus, *p < .001 vs. nonsocial stimulus. ***B***, Female C57BL/6J NS (*n* = 11) and PRS (*n* = 8) mice both demonstrated typical sociability behavior by showing a significant preference for a social stimulus over a nonsocial stimulus, *p < 0.001. ***C***, The same male C57BL/6J PRS mice exhibited significantly lower sociability compared to NS control mice, *p < 0.001. ***D***, Sociability index scores were comparable between the female NS and PRS C57BL/6J mice shown (in ***B***).

Sociability was assessed in NS and PRS mice as a representative measure of social ASD symptomatology. The findings of prenatal stress effects on sociability are shown in Figure 1. A two-way ANOVA of male NS and PRS sociability behavior revealed a significant stress condition × stimulus type interaction, *F*_(1,48)_ = 38.49, *p* = 1.221 × 10^−7^. Further, a significant main effect of stimulus type was found, *F*_(1,48)_ = 91.81, *p *= 1.006 × 10^−12^. Post hoc Tukey’s multiple comparisons tests revealed that male NS mice spent significantly more time interacting with the social stimulus (*M* = 98.42 s, SEM = 6.03) than the nonsocial stimulus (*M* = 27.85 s, SEM = 1.68), *p *= 4.230 × 10^−4^. In contrast, the difference between time spent interacting with the social (*M* = 71.03 s, SEM = 4.68) and nonsocial (*M* = 55.93 s, SEM = 4.35) stimuli was not significantly different in male PRS mice, *p *= 0.093 (Fig. 1*A*). The main effect of stress condition was found to be nonsignificant, *F*_(1,48)_ = 0.01, *p* = 0.939. An independent samples *t* test with a Welch’s correction for unequal variances revealed a significantly lower sociability index score for male PRS mice (*M* = 56.09, SEM = 2.68) than NS mice (*M* = 77.65, SEM = 1.22), *t*_(16.77)_ = 7.32, *p *= 1.307 × 10^−6^ (Fig. 1*B*). A separate two-way ANOVA revealed a significant main effect of stimulus type on duration of interaction (*F*_(1,36)_ = 116.30, *p *= 7.950 × 10^−13^), with both NS and PRS female mice spending a greater amount of time sniffing the social stimulus (*M* = 60.84 s, SEM = 5.40; *M* = 69.30 s, SEM = 4.39, respectively) than the nonsocial stimulus (*M* = 19.59 s, SEM = 3.49; *M* = 24.16 s, SEM = 2.38, respectively) (Fig. 1*C*). The main effect of stress condition (*F*_(1,36)_ = 2.64, *p *= 0.113) and stress condition × stimulus type interaction (*F*_(1,36)_ = 0.24, *p *= 0.630) in female mice was found to be nonsignificant. As shown in Figure 1*D*, an independent samples *t* test with a Welch’s correction for unequal variances indicated that the sociability index scores of female NS (*M* = 69.15, SEM = 3.20) and PRS mice (*M* = 75.19, SEM = 2.86) were not significantly different, *t*_(16.93)_ = 1.41, *p *= 0.177.

#### Reciprocal social interaction

**Figure 2.**
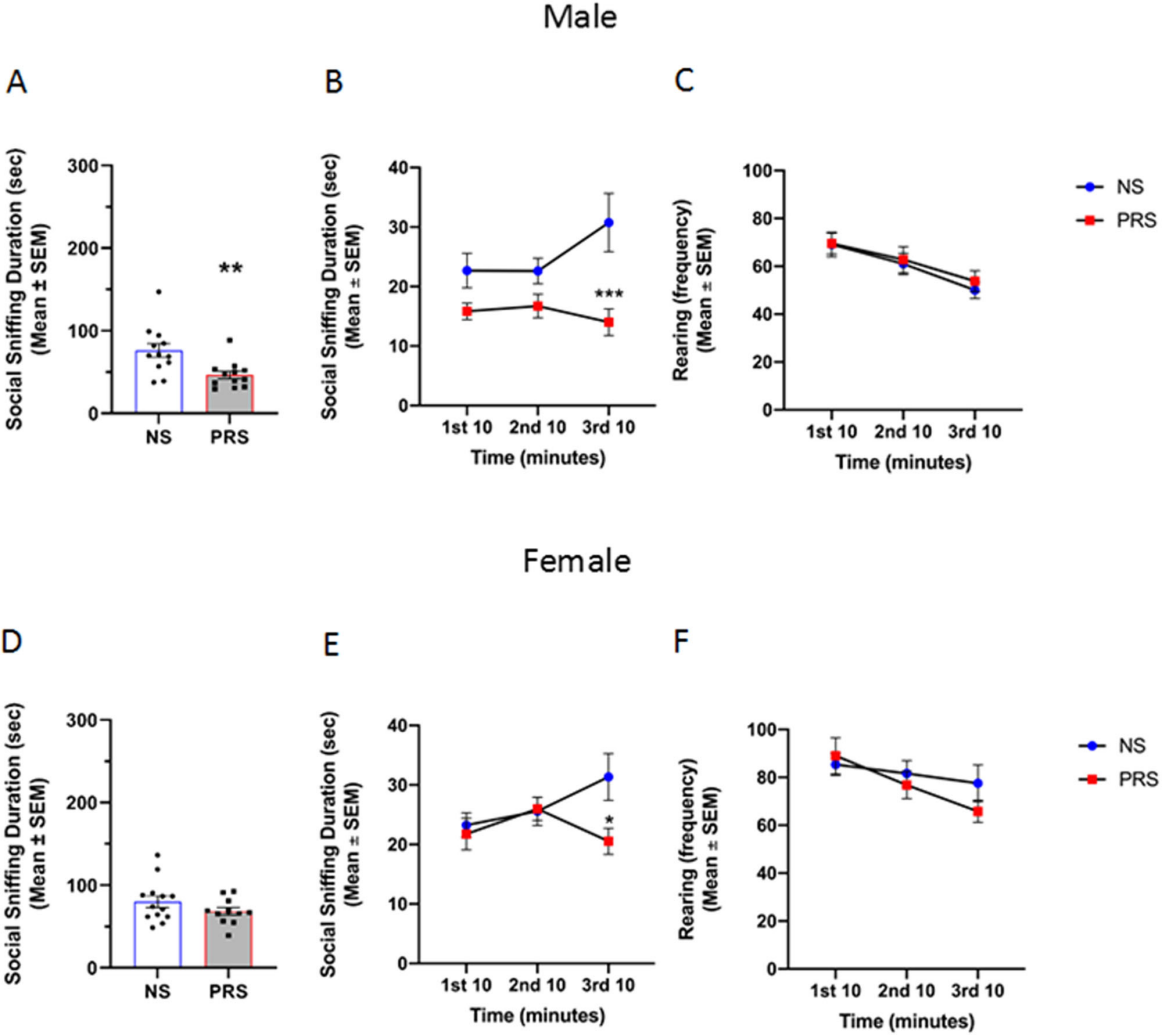
***A***, Male PRS (*n* = 12) mice engaged in significantly less social sniffing behavior than NS controls (*n* = 12), **p < 0.01. ***B***, The difference in cumulative social sniffing behavior between male NS and PRS mice appears to be driven by group differences in social sniffing during the final third of the testing session (***p < 0.001) which coincided with (***C***) decreased rearing behavior in both NS and PRS male mice across the testing session, *p *= 5.627 × 10^−7^. ***D***, Cumulative social sniffing behavior was not significantly lower in female PRS mice (*n* = 11) compared to NS controls (*n* = 13). ***E***, A difference in social sniffing behavior emerged during the final third of the reciprocal social interaction testing session, with female PRS mice exhibiting less social sniffing than NS female mice, *p < 0.05. F) Rearing behavior decreased in female NS and PRS mice across the reciprocal social interaction testing period, *p *= 0.001.

The effect of PRS on social behaviors pertinent to ASD was further investigated using the reciprocal social interaction test. The findings of prenatal stress on reciprocal social interaction are shown in Figure 2. An independent samples *t* test indicated that the cumulative duration of social sniffing in male PRS mice (*M* = 46.56 s, SEM = 4.67) was significantly lower than that observed in NS controls (*M* = 76.05 s, SEM = 8.51), *t*_(22)_ = 3.04, *p *= 0.006 (Fig. 2*A*). A repeated measures two-way ANOVA was conducted to evaluate male NS and PRS social sniffing across the testing session, and revealed a significant time × stress interaction for male social sniffing behavior, *F*_(2,44)_ = 4.36, *p *= 0.019. Further, a main effect of stress condition on social sniffing was detected, *F*_(1,22)_ = 9.23, *p *= 0.006. There was not a significant main effect of time on social sniffing behavior, *F*_(2,44)_ = 1.39, *p *= 0.261. Post hoc Tukey’s multiple comparisons tests indicated that social sniffing between male NS and PRS mice was comparable during the first 10 min (*p *= 0.249) and second 10 min (*p *= 0.375) of the testing session. However, PRS mice engaged in significantly less social sniffing (*M* = 14.00 s, SEM = 2.26) than NS mice (*M* = 30.74 s, SEM = 4.89) during the final 10 min of the testing session (*p *= 0.0003) (Fig. 2*B*). A coinciding main effect of time on rearing behavior during the reciprocal social interaction test was detected by a separate repeated measures two-way ANOVA, *F*_(2,44)_ = 20.32, *p *= 5.627 × 10^−7^ (Fig. 2*C*). There was not a significant main effect of stress condition on rearing behavior (*F*_(1,22)_ = 0.12, *p *= 0.732) or time × stress condition interaction, *F*_(2,44)_ = 0.19, *p *= 0.830.

**Figure 3.**
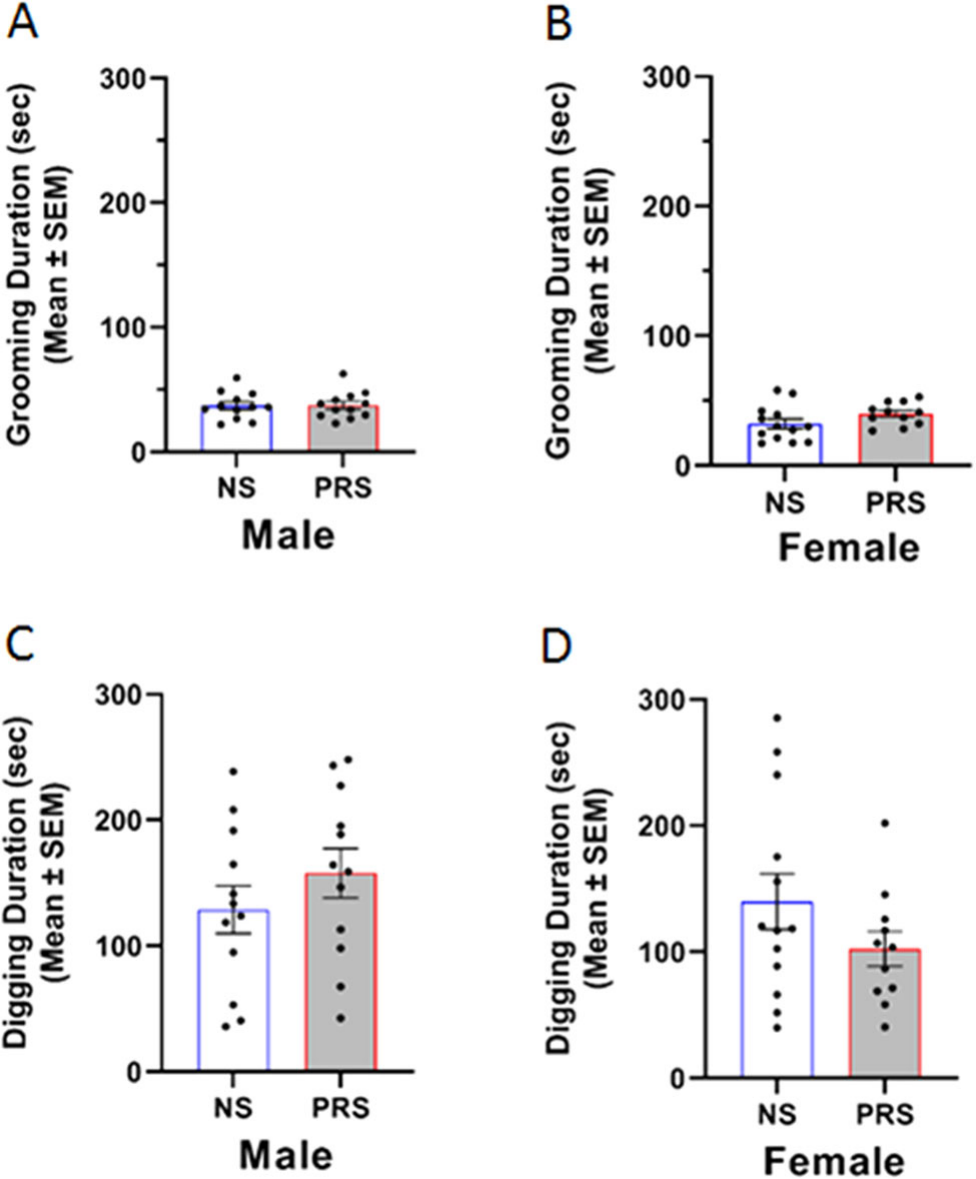
PRS mice did not engage in atypical levels of repetitive motor behaviors in the social context of the reciprocal social interaction behavior shown in Figure 2. ***A***, Male NS and PRS mice engaged in a similar amount of self-grooming behavior. ***B***, Female NS and PRS mice demonstrated comparable durations of self-grooming behavior. ***C***, Male NS and PRS mice were additionally found to perform comparable amounts of digging behavior. ***D***, Differences in digging behavior were not observed between female NS and PRS mice.

An independent samples *t* test indicated that the difference in cumulative duration of social sniffing between female NS (*M* = 80.19 s, SEM = 7.01) and PRS mice (*M* = 68.27 s, SEM = 4.71) was nonsignificant, *t*_(22)_ = 1.36, *p *= 0.188 (Fig. 2*D*). However, a repeated measures two-way ANOVA indicated a significant time × stress condition interaction *F*_(2,44)_ = 4.13, *p *= 0.023. Main effects of time and stress condition on social sniffing were not found to be significant, *F*_(2,44)_ = 1.72, *p* = .191; *F*_(1,22)_ = 1.84, *p *= 0.188, respectively. Post hoc Tukey’s multiple comparisons tests revealed that female NS and PRS social sniffing was not significantly different during the first 10 min (*p *= 0.971) or second 10 min (*p *> 0.999) of the testing session. However, female PRS mice engaged in significantly less social sniffing behavior (*M* = 20.54 s, SEM = 2.17) than NS controls (*M* = 31.35 s, SEM = 3.93) during the final 10 min of the testing session (*p *= 0.017) (Fig. 2*E*). A coinciding main effect of time on rearing behavior during the reciprocal social interaction test was detected by a separate repeated measures two-way ANOVA, *F*_(2,44)_ = 7.69, *p *= 0.001 (Fig. 2*F*). There was not a significant main effect of stress condition on rearing behavior (*F*_(1,22)_ = 0.35, *p *= 0.563) or time × stress condition interaction, *F*_(2,44)_ = 1.88, *p *= 0.164. The findings of the effect of prenatal stress exposure on nonsocial/repetitive behavior in a social context are represented in Figure 3. An independent samples *t* test indicated that male PRS mice (*M* = 37.46 s, SEM = 3.13) did not engage in a significantly greater amount of self-grooming behavior compared to NS controls (*M* = 37.02 s, SEM = 3.15) during the reciprocal social interaction assay, *t*_(22)_ = 0.10, *p *= 0.923 (Fig. 3*A*). Similarly, there was no significant difference between male NS (*M* = 128.80 s, SEM = 18.92) and PRS (*M* = 157.90 s, SEM = 19.43) digging behavior during the reciprocal social interaction assay, *t*_(22)_ = 1.07, *p *= 0.295 (Fig. 3*B*). An independent samples *t* test indicated that female PRS mice (*M* = 39.96 s, SEM = 2.61) did not engage in a significantly greater amount of self-grooming behavior compared to NS controls (*M* = 32.20 s, SEM = 3.78) during the reciprocal social interaction assay, *t*_(22)_ = 1.63, *p *= 0.118 (Fig. 3*C*). Similarly, there was no significant difference between female NS (*M* = 140.00 s, SEM = 21.99) and PRS (*M* = 102.40 s, SEM = 13.70) digging behavior during the reciprocal social interaction assay, *t*_(22)_ = 1.39, *p *= 0.179 (Fig. 3*D*).

#### Self-grooming behavior

**Figure 4.**
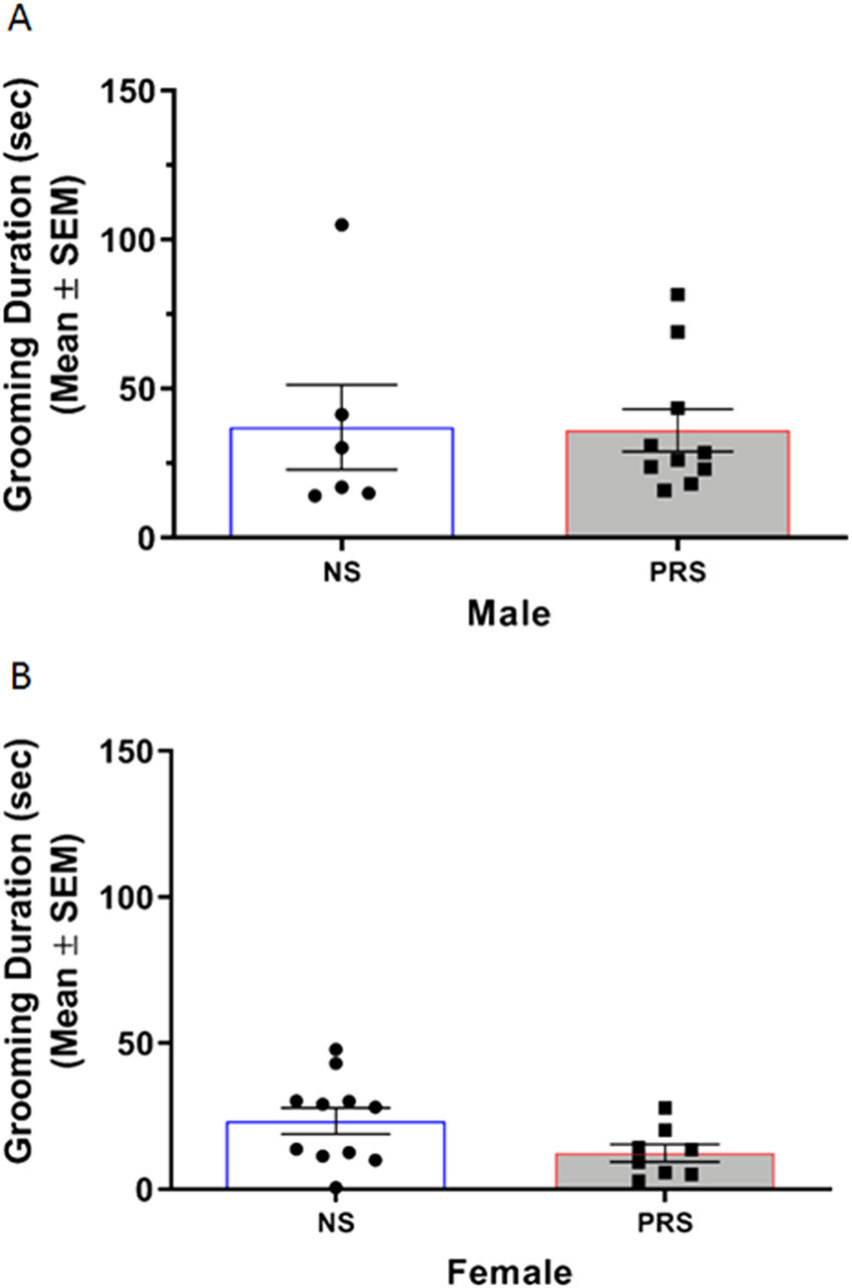
***A***, Male PRS mice did not demonstrate stereotyped motor behavior in the form of self-grooming. The duration of self-grooming behavior was comparable between male NS (*n* = 6) and PRS (*n* = 10) mice. ***B***, Similar levels of self-grooming behavior were additionally found in female NS (*n* = 11) and PRS (*n* = 8) mice.

Self-grooming was measured as a representative behavior for the RRBs characteristic of ASD. Figure 4 depicts the self-grooming behavior of NS and PRS mice. An independent samples *t* test indicated that male PRS mice (*M* = 27.65 s, SEM = 6.66) did not engage in a significantly greater amount of self-grooming behavior compared to NS controls (*M* = 28.12 s, SEM = 6.54), *t*_(23)_ = 0.05, *p *= 0.962 (Fig. 4*A*). A separate independent samples *t* test indicated that female PRS mice (*M* = 21.89 s, SEM = 2.28) did not engage in a significantly greater amount of self-grooming behavior compared to NS controls (*M* = 20.82 s, SEM = 3.51), *t*_(18)_ = 0.26, *p *= 0.796 (Fig. 4*B*).

#### Marble burying behavior

**Figure 5.**
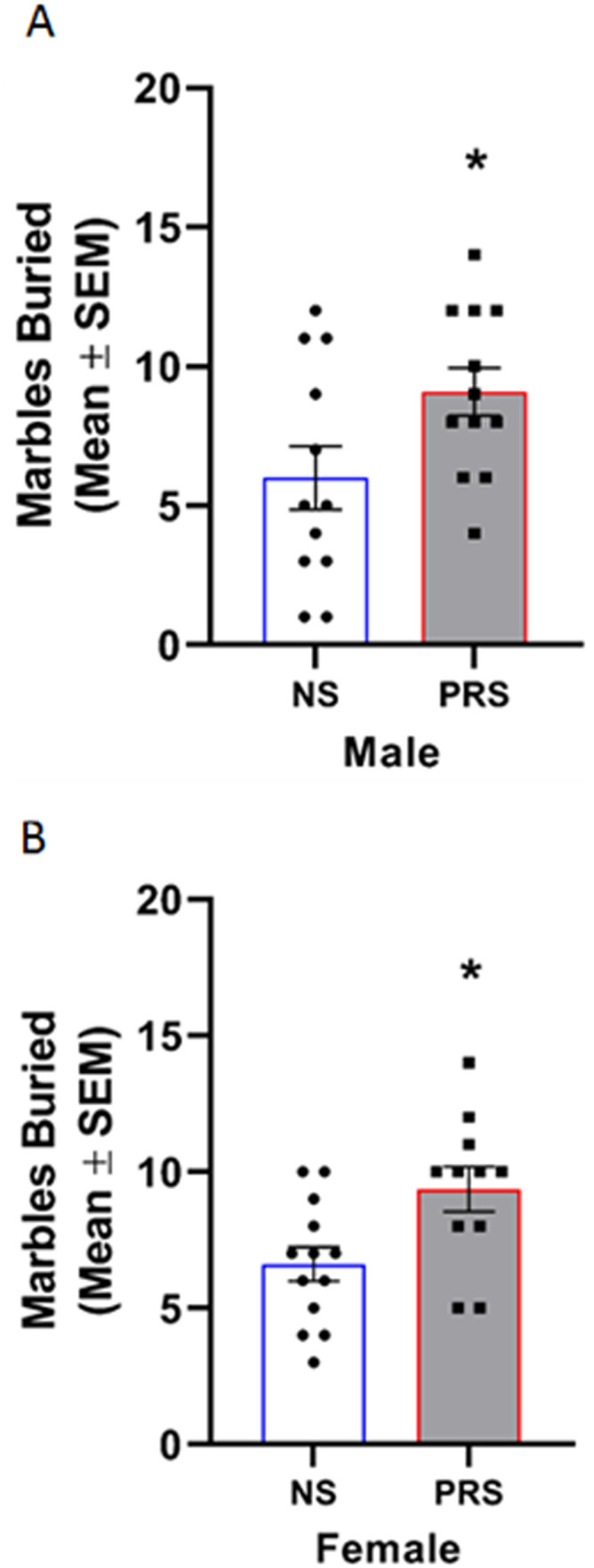
PRS mice (*n* = 12) demonstrated a motor RRB in the form of increased marble burying behavior relative to that observed in NS mice (*n* = 12). ***A***, Male PRS mice displayed an increase in marble burying behavior compared to NS controls. ***B***, Female PRS mice (*n* = 11) buried a higher number of marbles than female NS mice (*n* = 13). *p < 0.05.

Marble burying was additionally measured as a representative behavior for RRBs. Marble burying behavior of NS and PRS mice is shown in Figure 5. An independent samples *t* test indicated that male PRS mice (*M* = 9.08 marbles buried, SEM = 0.87) engaged in a significantly greater amount of marble burying behavior compared to NS controls (*M* = 6.00 marbles buried, SEM = 1.14), *t*_(22)_ = 2.16, *p *= 0.042 (Fig. 5*A*). A separate independent samples *t* test similarly indicated that female PRS mice (*M* = 9.36 marbles buried, SEM = 0.82) buried a significantly greater number of marbles compared to NS controls (*M* = 6.62 marbles buried, SEM = 0.63), *t*_(22)_ = 2.70, *p *= 0.013 (Fig. 5*B*).

#### Elevated plus maze

**Figure 6.**
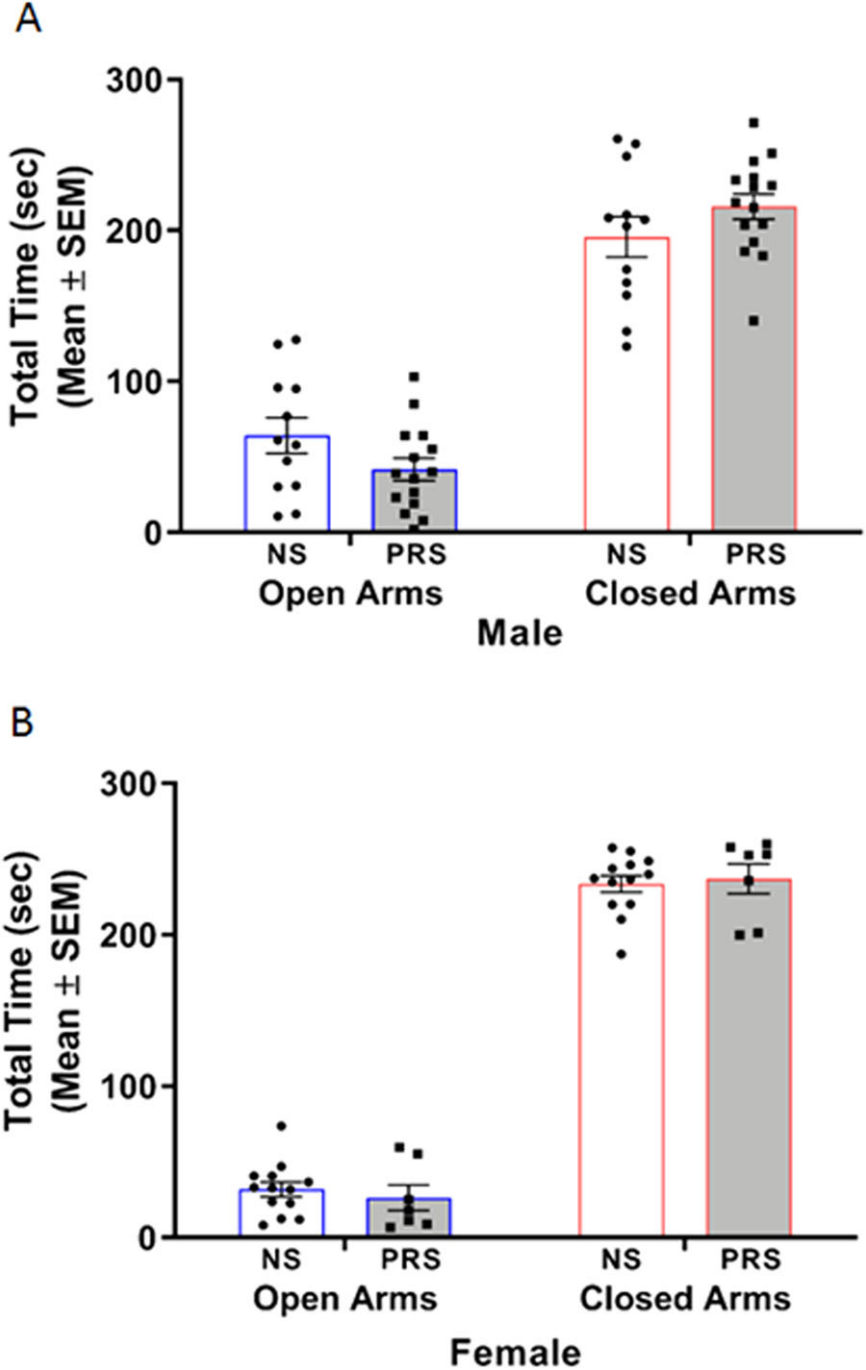
The presence of anxiety-like behavior was not detected in PRS mice through use of the EPM. ***A***, Differences in the amount of time spent in the open and closed arms of the EPM were nonsignificant in male NS (*n* = 12) and PRS (*n* = 15) mice. ***B***, Female NS (*n* = 13) and PRS (*n* = 7) mice also spent a comparable amount of time in the open and closed arms of the EPM, respectively.

EPM testing was performed to assess whether PRS exposure promoted anxiety-like behavior. A two-way ANOVA of male C57BL/6J behavior in the EPM indicated a significant stress condition × arm type interaction *F*_(1,50)_ = 4.43, *p *= 0.040. However, post hoc Tukey’s multiple comparisons tests showed that male NS and PRS mice spent a comparable amount of time in both the open (*M* = 64.14 s, SEM = 11.68; *M* = 41.78 s, SEM = 7.43, respectively) (*p *= 0.407) and closed arms (*M* = 195.72 s, SEM = 13.34; *M* = 215.83 s, SEM = 8.40, respectively) (*p *= 0.500) of the EPM during the 5 min testing session (Fig. 6*A*). The two-way ANOVA additionally indicated a main effect of arm type on time spent in arms, *F*_(1,50)_ = 229.30, *p* < 1.000 × 10^−15^. No main effect of stress condition on time spent in arms was found, *F*_(1,50)_ = 0.01, *p *= 0.912. A separate two-way ANOVA of female C57BL/6J behavior in the EPM indicated a nonsignificant stress condition × arm type interaction, *F*_(1,36)_ = 0.44, *p *= 0.511. There was a significant main effect of arm type on time spent in arms (*F*_(1,36)_ = 908.60, *p *< 1.000 × 10^−15^), with both NS and PRS female mice spending more time in the closed arms (*M* = 233.55 s, SEM = 5.45; *M* = 237.07 s, SEM = 9.92, respectively) than the open arms (*M* = 31.84  s, SEM = 4.84; *M* = 26.29 s, SEM = 8.34, respectively) of the EPM (Fig. 6*B*). There was not a main effect of stress condition on time spent in arms, *F*_(1,36)_ = 0.02, *p *= 0.880.

#### Light/dark box

**Figure 7.**
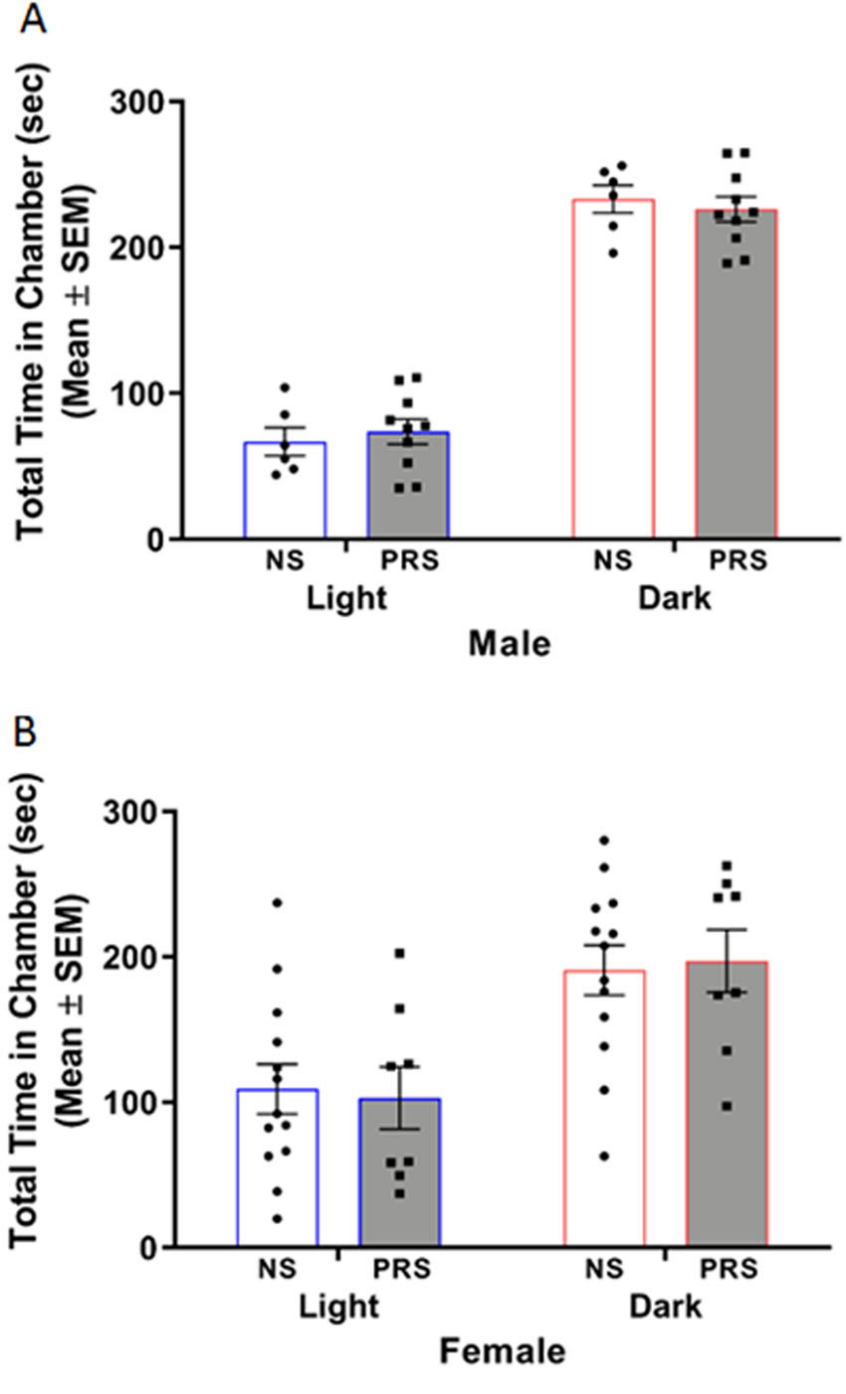
***A***, Male NS (*n* = 6) and PRS (*n* = 10) mice did not significantly differ in the amount of time spent in the light and dark compartments of the light/dark box used to investigate anxiety-like behavior. ***B***, Female PRS (*n* = 8) mice spent a similar amount of time in the light and dark compartments of the light/dark box as NS controls (*n* = 13), respectively.

The potential relationship between PRS exposure and anxiety-like behavior was additionally investigated through implementation of the light/dark box. A two-way ANOVA of male C57BL/6J behavior in the light/dark box indicated a nonsignificant stress condition × chamber type interaction, *F*_(1,28)_ = 0.55, *p *= 0.465. There was a significant main effect of chamber type on time spent in chamber (*F*_(1,28)_ = 288.70, *p *< 1.000 × 10^−15^), with both NS and PRS male mice spending more time in the dark chamber (*M* = 233.09 se SEM = 9.51; *M* = 226.15 s, SEM = 8.50, respectively) than the light chamber (*M* = 66.80 s, SEM = 9.54; *M* = 73.76 s, SEM = 8.51, respectively) of the light/dark box (Fig. 7*A*). There was not a main effect of stress condition on time spent in chamber, *F*_(1,28)_ = 7.58 × 10^−7^, *p *> 0.999. A separate two-way ANOVA of female C57BL/6J behavior in the light/dark box indicated a nonsignificant stress condition × chamber type interaction, *F*_(1,38)_ = 0.10, *p *= 0.750. There was a significant main effect of chamber type on time spent in chamber (*F*_(1,38)_ = 20.40, *p *= 5.932 × 10^−5^), with both NS and PRS female mice spending more time in the dark chamber (*M* = 190.85 s, SEM 17.12= ; *M* = 197.10 s, SEM = 21.45, respectively) than the light chamber (*M* = 109.07 s, SEM = 17.12; *M* = 102.82 s, SEM = 21.45, respectively) of the light/dark box (Fig. 7*B*). There was not a main effect of stress condition on time spent in chamber, *F*_(1,38)_ = 1.26 × 10^−8^, *p *> 0.999.

#### Locomotor activity

**Figure 8.**
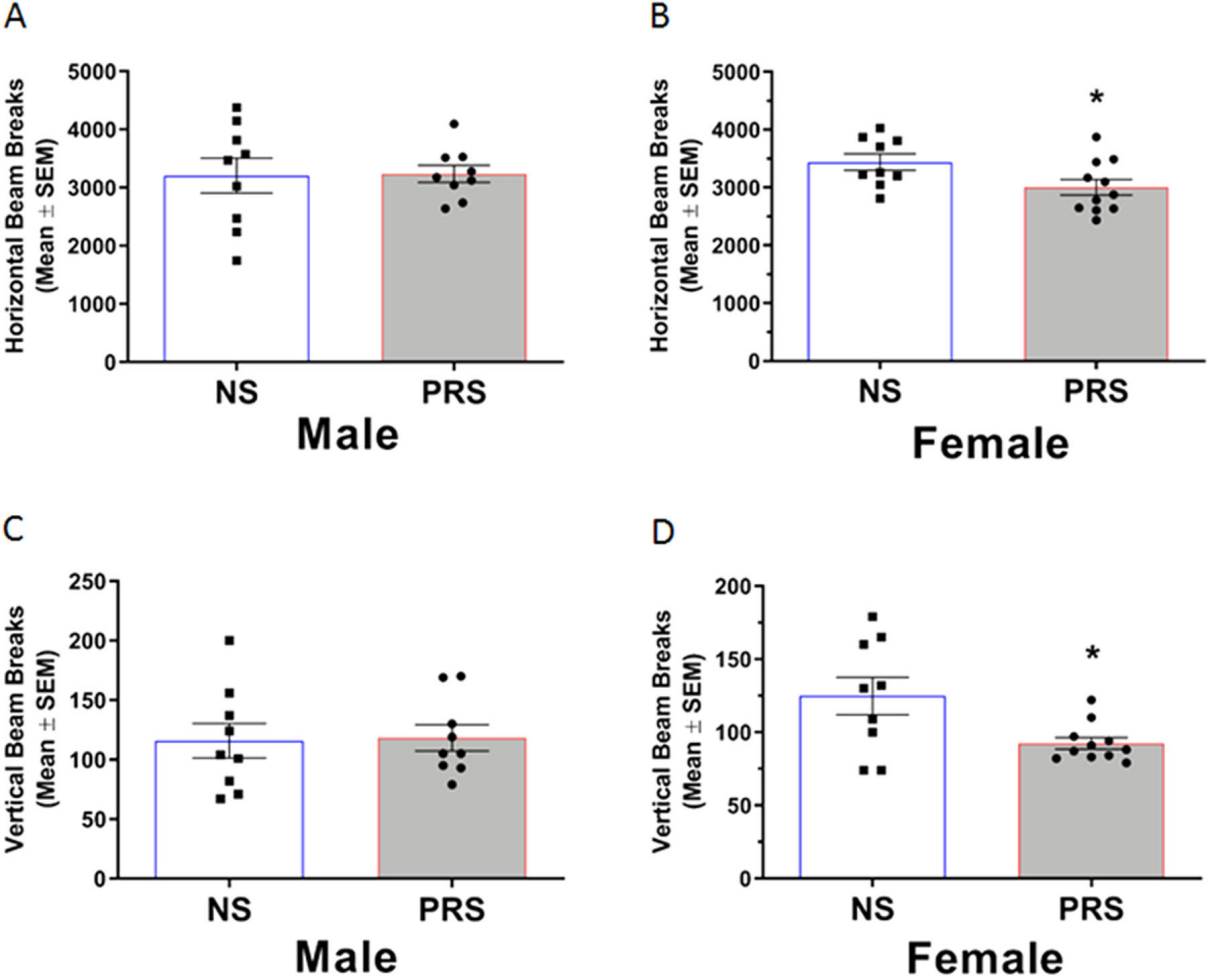
***A***, Male NS (*n* = 9) and PRS (*n* = 9) mice exhibited a similar level of locomotor activity as measured by horizontal beam breaks in an open field. ***B***, Female PRS mice (n = 11) were found to be hypoactive compared to NS mice (*n* = 9) based on the breaking of significantly fewer horizontal beams in an open field apparatus. ***C***, The number of vertical beam breaks by the same male PRS mice shown (in ***A***) did not significantly differ from the vertical activity (i.e., beam breaks) of NS mice. ***D***, Measurement of vertical beam breaks in an open field additionally indicated reduced activity in the same female PRS mice shown (in ***B***) compared to NS controls. *p < 0.05.

Measurement of locomotor activity was performed to determine whether more general motor differences might underlie the presence of RRB-like behavior in PRS mice. Locomotor activity in NS and PRS mice is shown in Figure 8. An independent samples *t* test indicated that male NS (*M* = 3206.00 beam breaks, SEM = 300.10) and PRS mice (*M* = 3237.00 beam breaks, SEM = 146.90) exhibited a similar level of horizontal activity, *t*_(16)_ = 0.09, *p *= 0.927 (Fig. 8*A*). Similarly, vertical activity was not significantly different between male NS (*M* = 115.80 beam breaks, SEM = 14.49) and PRS mice (*M* = 118.30 beam breaks, SEM = 10.84), *t*_(16)_ = 0.14, *p *= 0.889 (Fig. 8*B*). However, independent samples *t* tests showed lower locomotor activity in female PRS mice compared to NS controls as measured by horizontal beam breaks (*M* = 3,005.00, SEM = 135.90; *M* = 3,439.00, SEM = 141.00, respectively, *t*_(18)_ = 2.21, *p *= 0.041) and vertical beam breaks (*M* = 92.45, SEM = 3.94; *M* = 124.80, SEM = 12.85, respectively, *t*_(9.51)_ = 2.40, *p *= 0.038) in an open field (Fig. 8*C,D*). A Welch’s correction for unequal variances was applied to the *t* test of vertical beam breaks by female NS and PRS mice.

### Investigation of epigenetic alterations in PRS mice through behavioral pharmacology

#### NS and PRS mouse three-chamber sociability

**Figure 9.**
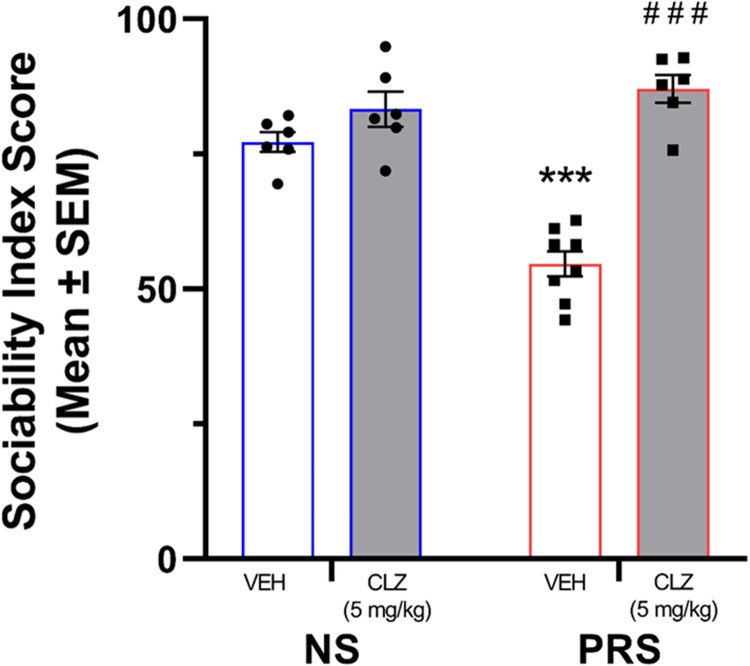
VEH-treated PRS mice (*n* = 8) exhibited reduced sociability compared to VEH-treated NS mice (*n* = 6). The sociability deficit observed in VEH-treated PRS mice was rescued by CLZ treatment (5 mg/kg) – (PRS-CLZ *n* = 6, NS-CLZ *n* = 6). ***p < 0.001 vs. VEH-treated NS mice, ^###^*p *< 0.001 vs. VEH-treated PRS mice.

A two-way ANOVA was conducted to evaluate the effect of CLZ as a pharmacotherapy with epigenetic properties on male NS and PRS mouse sociability (Fig. 9). Results of the ANOVA indicated a significant effect of treatment × stress condition interaction on sociability behavior, *F*_(3,39)_ = 10.63, *p *= 3.043 × 10^−5^. Further, a significant main effect of treatment on sociability was found (*F*_(3,39)_ = 17.89, *p *= 1.841 × 10^−7^). However, the main effect of stress condition on sociability fell short of statistical significance, *F*_(3,39)_ = 3.48, *p *= 0.070. Post hoc Tukey’s multiple comparisons showed that sociability index scores of male VEH-treated PRS mice (*M* = 54.60, SEM = 2.34) were significantly lower than in VEH-treated NS controls (*M* = 77.23, SEM = 1.83), *p *= 1.160 × 10^−5^. In addition, the sociability index scores of PRS mice treated with CLZ (*M* = 87.02, SEM = 2.60) were significantly higher than those of VEH-treated PRS mice, *p *= 1.380 × 10^−4^.

#### Marble burying

**Figure 10.**
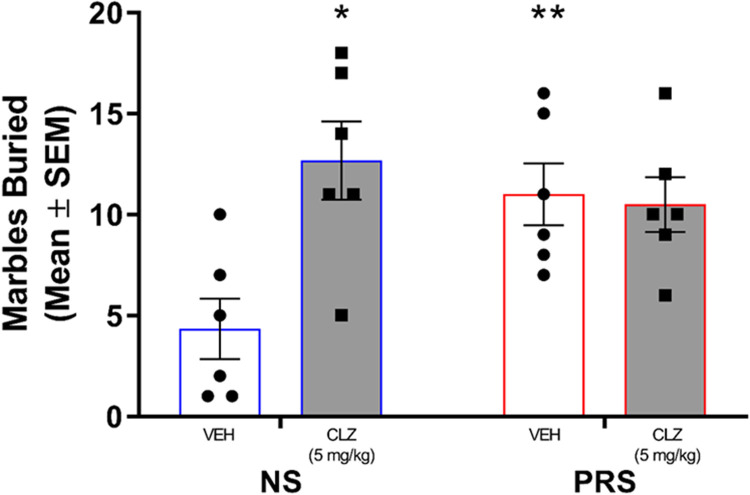
Male VEH-treated PRS mice (*n* = 6) buried significantly more marbles than VEH-treated NS mice (*n* = 6). CLZ (5 mg/kg) treatment did not attenuate high levels of marble burying in PRS mice (*n* = 6), but rather increased marble burying behavior in NS mice (*n* = 6). *p < 0.05 vs. VEH-treated NS, **p < 0.01 vs. VEH-treated NS.

A two-way ANOVA was conducted to evaluate the effect of CLZ treatment on male NS and PRS mouse marble burying behavior (Fig. 10). Results of the ANOVA indicated a significant effect of treatment × stress condition interaction on marble burying behavior, *F*_(1,20)_ = 7.65, *p *= 0.012. Further, a significant main effect of treatment on marble burying was found (*F*_(1,20)_ = 6.01, *p *= 0.024). The main effect of stress condition on marble burying was not statistically significant, *F*_(1,20)_ = 1.98, *p *= 0.174. Post hoc Tukey’s multiple comparisons showed that the number of marbles buried by male VEH-treated PRS mice (*M* = 11.00, SEM = 1.53) was significantly higher than that of the VEH-treated NS controls (*M* = 4.33, SEM = 1.50), *p *= 0.036. The number of marbles buried by PRS mice that received VEH treatment and CLZ-treated PRS mice (*M* = 10.50, SEM = 1.36) was not significantly different, *p *= 0.996. However, CLZ-treated NS mice buried a significantly higher number of marbles (*M* = 12.67, SEM = 1.94) than VEH-treated NS mice (*p *= 0.007), which was comparable to the marble burying of VEH- treated PRS mice (*p *= 0.881) and CLZ-treated PRS mice (*p *= 0.774).

### Investigation of excitation-inhibition balance in social and repetitive behavior neurocircuitry by qRT-PCR

**Figure 11.**
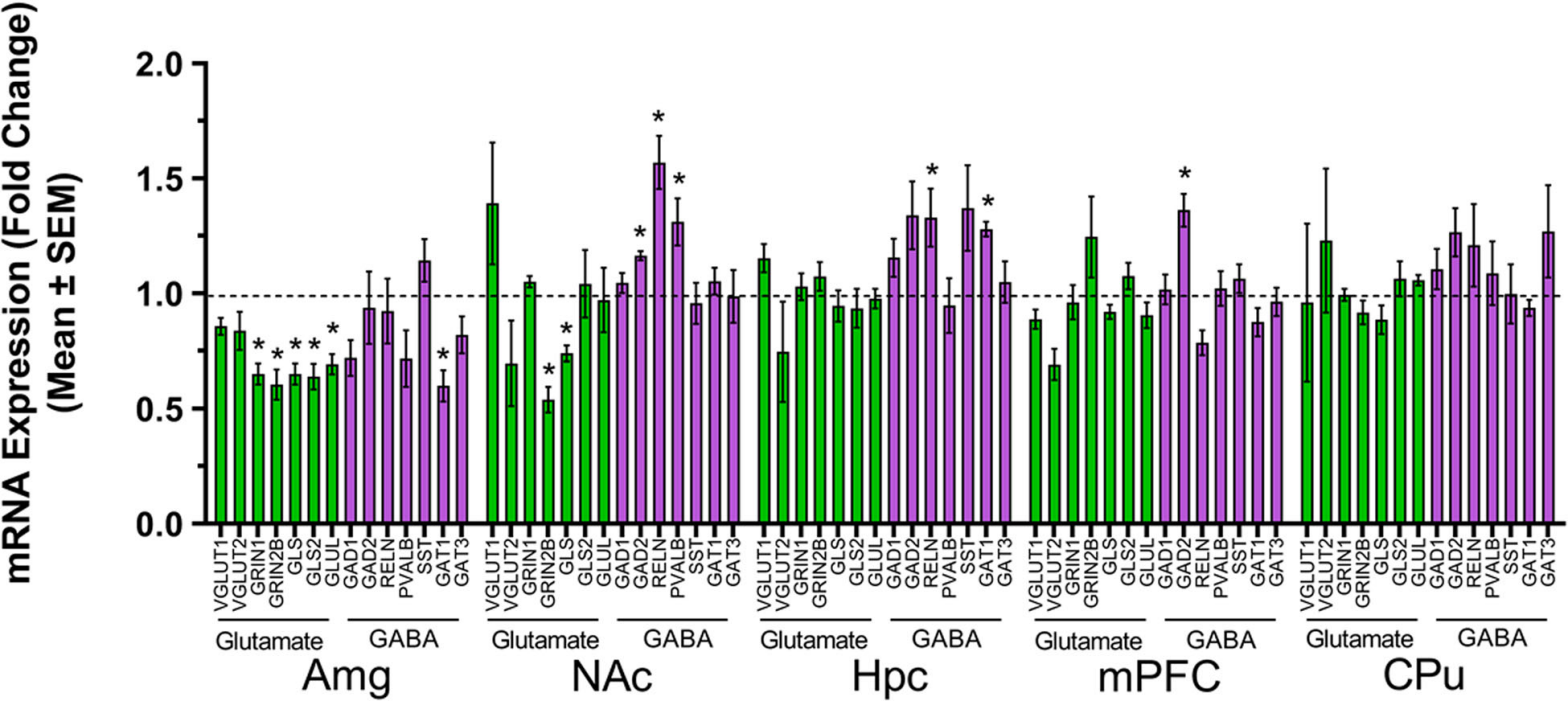
Aberrant expression levels of glutamatergic (green) and GABAergic (purple) gene markers were detected in the male PRS (*n* = 5) brain relative to NS controls (*n* = 7) by qRT-PCR. In the Hpc, Reln and Gat1 were, respectively, more highly expressed in PRS mice than NS mice. The expression levels of Grin2b and Gls were each lower in the PRS NAc than NS NAc. Higher levels of Gad2, Reln, and Pvalb were detected in the PRS NAc relative to the expression observed in the NAc of NS mice. Decreased expression levels of Grin1, Grin2b, Gls, Gls2, Glul, and Gat1 were found in the Amg of PRS mice compared to NS controls. Gad2 expression was higher in the mPFC of PRS mice relative to NS mice. All bars represent mRNA levels corresponding to PRS mice. NS mRNA levels, to which measurements of PRS gene expression were normalized, is represented by the dashed line for each respective gene. *p < 0.05. For additional information related to the data presented here, please see Extended Data Table 11-1.

All results from separate independent samples *t* tests performed on data from individual qRT-PCR experiments measuring the relative expression levels of GOI in samples derived from NS and PRS mouse amygdala (Amg), nucleus accumbens (NAc), medial prefrontal cortex (mPFC), hippocampus (Hpc), and caudate putamen (CPu) are depicted in Figure 11.

#### Amygdala

Aberrant expression of the glutamatergic gene markers *Grin1* (*t*_(10)_ = 4.43, *p *= 0.001), *Grin2b* (*t*_(10)_ = 4.33, *p *= 0.002), *Gls* (*t*_(10)_ = 5.94, *p *= 0.0001), *Gls2* (*t*_(10)_ = 3.91, *p *= 0.003), and *Glul* (*t*_(10)_ = 3.20, *p *= 0.010) was revealed in the PRS Amg by separate independent samples *t* tests. Further, the expression levels of *Grin1* (*M*_FC _= 0.65, SEM = 0.05), *Grin2b* (*M*_FC _= 0.61, SEM = 0.07), *Gls* (*M*_FC _= 0.65, SEM = 0.05), *Gls2* (*M*_FC _= 0.64, SEM = 0.06), and *Glul* (*M*_FC _= 0.69, SEM = 0.04) were each lower in PRS mice relative to NS mice (*M_Grin1_*_ FC _= 1.00, SEM = 0.06; *M_Grin2b_*_ FC _= 1.00, SEM = 0.06; *M_Gls_*_ FC _= 1.00, SEM = 0.04; *M_Gls2_*_ FC_ = 1.00, SEM = 0.07; *M_Glul_*_ FC_ = 1.00, SEM = 0.07). Expression levels of the GABAergic gene marker *Gat1* were also significantly lower in the Amg of PRS mice (*M*_FC _= 0.60, SEM = 0.07) compared to NS controls (*M*_FC _= 1.00, SEM = 0.11), *t*_(10)_ = 2.81, *p *= 0.018.

#### Nucleus accumbens

Results of separate independent samples *t* tests indicated significant differences in the fold change (FC) expression levels of *Grin2b* (*t*_(7.09)_ = 2.41, *p *= 0.047), *Gls* (*t*_(10)_ = 2.54, *p *= 0.029), *Gad2* (*t*_(10)_ = 2.64, *p *= 0.025), *Reln* (*t*_(10)_ = 2.95, *p *= 0.015), and *Pvalb* (*t*_(9)_ = 2.70, *p *= 0.024) in the NAc of male PRS mice compared to WT controls. A Welch’s correction for unequal variances was applied to the analysis of *Grin2b*. More specifically, the expression levels of the glutamatergic gene markers *Grin2b* (M _FC_ = 0.54, SEM = 0.06) and *Gls* (*M*_FC_ = 0.74, SEM = 0.03) were significantly lower in PRS mice than in NS mice (*M_Grin2b_*_ FC_ = 1.00, SEM = 0.18; *M_Gls_*_ FC_ = 1.00, SEM = 0.08), whereas the PRS expression levels of the GABAergic marker genes *Gad2* (*M*_FC_ = 1.16, SEM = 0.02), *Reln* (*M*_FC_ =1.57, SEM = 0.12), and *Pvalb* (*M*_FC_ = 1.31, SEM = 0.10) were significantly higher than those detected in the NS NAc, *M_Gad2_*_FC _= 1.00, SEM = 0.05; *M_Reln_*_FC_ = 1.00, SEM = 0.14; *M_Pvalb_*_FC_ = 1.00, SEM = 0.06.

#### Hippocampus, medial prefrontal cortex, and caudate putamen

Respective independent *t* tests revealed relatively few differentially expressed glutamatergic and GABAergic marker genes in the Hpc, mPFC, and CPu of PRS mice compared to NS mice. In fact, only differences in GABAergic marker genes were detected between NS and PRS mice in the Hpc and mPFC, and no significant changes in expression level were detected in the PRS CPu. In the Hpc, *Reln* (*M*_FC _= 1.33, SEM = 0.13) and *Gat1* (*M* = 1.28, SEM = 0.03) expression levels were higher in PRS mice than in NS mice (*M_Reln_*_ FC _= 1.00, SEM = 0.07; *M_Gat1_*_ FC _= 1.00, SEM = 0.03), *t*_(10)_ = 2.44, *p *= 0.035; *t*_(9)_ = 5.35, *p *= 0.0005, respectively. In the mPFC, *Gad2* expression levels were significantly higher in PRS mice (*M*_FC_ = 1.36, SEM = 0.11) than in NS controls (*M*_FC _= 1.00, SEM = 0.06), *t*_(9)_ = 3.13, *p *= 0.012. As presented in detail in Table 11-1, all other changes in glutamatergic and GABAergic marker gene expression were statistically nonsignificant.

### RNA fluorescent barcoding investigation of an epigenetic basis for the PRS behavioral phenotype and clozapine action in the nucleus accumbens

Differential expression of epigenetic enzymes in the NAc of NS and PRS mice receiving vehicle and CLZ treatments was revealed by separate two-way ANOVAs. As shown in Table 1, a main effect of CLZ was detected for the number of normalized transcript counts of 11 genes with putative roles in epigenetics (DNA methylation = 1, histone acetylation = 4, histone methylation = 6) at the significance threshold of FDR < 0.05; a higher number of transcript counts was detected in CLZ-treated mice than VEH-treated mice for all 11 genes (Kdm4a, *Kmt2b, Kmt2c, Phf2, Setdb1, Smyd3, Mecp2, Hdac5, Hdac6, Kat8, Rela*). Main effects of stress condition were nonsignificant at FDR < 0.05 for all selected GoI (Extended Data Table 1-2). Stress condition × treatment interaction effects were also nonsignificant for all selected GoI at FDR < 0.05.

**Table 1.** Treatment with CLZ (5 mg/kg, twice daily for 5 d + 18 hr washout) increased the expression of 11 genes in the nucleus accumbens pertinent to the epigenetic processes of DNA methylation, histone methylation, and histone acetylation in both NS and PRS mice, FDR < 0.05.

Gene	Effect	*P* value	FDR	Fold change	Mechanistic involvement
*Mecp2*	Main effect CLZ	8.1930 × 10^−6^	0.0008	1.19	DNA methylation
*Kmt2c*	Main effect CLZ	5.3504 × 10^−6^	0.0008	1.15	Histone methylation
*Phf2*	Main effect CLZ	4.3315 × 10^−5^	0.0023	1.17	Histone methylation
*Setdb1*	Main effect CLZ	0.0002	0.0079	1.22	Histone methylation
*Smyd3*	Main effect CLZ	0.0005	0.0156	1.23	Histone methylation
*Kdm4a*	Main effect CLZ	0.0006	0.0159	1.19	Histone methylation
*Kmt2b*	Main effect CLZ	0.0018	0.0442	1.22	Histone methylation
*Nfkb* (*Rela*)	Main effect CLZ	3.5653 × 10^−5^	0.0023	1.32	Histone acetylation
*Hdac5*	Main effect CLZ	4.6193 × 10^−5^	0.0023	1.17	Histone acetylation
*Hdac6*	Main effect CLZ	0.0001	0.0058	1.14	Histone acetylation
*Kat8*	Main effect CLZ	0.0002	0.0068	1.13	Histone acetylation

For additional data related to the information presented in Table 1, please see Extended Data Tables 1-1 and 1-2.

10.1523/ENEURO.0186-23.2024.t1-1 Table 1-1 Full custom gene panel assessed for effects of prenatal stress exposure, CLZ treatment, and their interaction via RNA fluorescent barcoding (NanoString Technologies). Download Table 1-1, DOCX file.

10.1523/ENEURO.0186-23.2024.t1-2 Table 1-2 Expanded RNA fluorescent barcoding results of interaction and main effects for prenatal stress exposure and CLZ treatment the in the NS and PRS NAc. Download Table 1-2, DOCX file.

10.1523/ENEURO.0186-23.2024.t11-1 Table 11-1 Results of nonsignificant differences in mRNA expression levels of glutamatergic and GABAergic gene markers in the Hpc, NAc, Amg, mPFC, and CPu of NS and PRS mice presented in Figure 11. Download Table 11-1, DOCX file.

## Discussion

Maternal exposure to stress-inducing events during pregnancy is associated with fetal neurodevelopment being steered toward adverse psychiatric outcomes, including ASD (Ward, 1990; Beversdorf et al., 2005; Kinney et al., 2008). However, the precise neurobiological underpinnings of the relationship between chronic prenatal stress exposure and the development of ASD symptoms are incompletely understood. Moreover, there exists a particular paucity of studies on the effects of prenatal stress on the C57BL/6J genetic background, which is commonly used for the generation of genetic mouse models for the study of ASD. The present study addresses this gap in the preclinical ASD literature through behavioral and molecular characterizations that allow for the effects of prenatal stress exposure to be contextualized amongst other mouse models for the study of ASD.

The marble burying results of the present experiments are the first report of an RRB presenting in both male and female C57BL/6J PRS mice. Measurement of horizontal and vertical locomotor activity in an open field apparatus indicated that the high level of marble burying behavior in PRS mice was not explained by hyperactivity in male or female PRS mice more broadly. To the contrary, female PRS mice were found to be hypoactive relative to NS controls as measured by horizontal and vertical beam breaks, respectively. In the present experiments, there were no differences in anxiety-like behaviors between male NS and PRS mice or female NS and PRS mice in the light/dark box, or EPM. As such, the finding of increased marble burying is interpreted here as an increase in compulsive-like motor behavior.

In agreement with our self-grooming findings, Matsui et al. (2018) similarly report self-grooming behavior to be comparable between NS and PRS mice generated on the C57BL/6J genetic background. Interestingly, however, male heterozygous SERT knockout mice generated on the C57BL/6J genetic background and exposed to prenatal stress exhibit a higher average duration of self-grooming bouts than controls (Matsui et al., 2018). Therefore, whereas exposure to prenatal stress appears to confer some level of risk for the development of a motor RRB, such a behavioral endophenotype may be exacerbated by specific gene-environment interactions. In line with the two-hit hypothesis of ASD (Millan, 2013), the degree to which an environmental insult impacts behavior may well be related to individual genetic vulnerabilities and subsequent epigenetic modifications.

Regarding social behavior, male PRS mice demonstrated lower sociability than NS control mice in the three-chamber test of sociability. This evidence of a sociability deficit in male PRS mice was consistent with the finding of reduced social interaction in male C57BL/6J PRS mice by Gur et al. (2019). The presence of atypical social behavior in PRS mice was further substantiated in the current work by the finding of decreased social sniffing behavior in PRS mice during the reciprocal social interaction assay. Analysis of both social (e.g., social sniffing) and nonsocial (e.g., rearing) behaviors across the 30 min testing session showed that the emergence of atypical social behavior in the reciprocal social interaction assay was most readily detectable in PRS mice during an extended testing session. The amount of social sniffing demonstrated by NS and PRS mice during the first and second 10 min periods of the testing session was not significantly different in either male or female mice. A social deficit only became apparent in PRS mice during the final 10 min of testing. This pattern of social sniffing behavior coincided with a decrease in rearing across the testing session in both sexes of NS and PRS mice. The decline in frequency of rearing behavior across the testing session reflected a reduction in exploratory behavior as the testing arena became less novel over time. In essence, the first two-thirds of the testing session may have served as a de facto habituation period during which a preoccupation with exploration of the novel environment masked the differences in social sniffing behavior between NS and PRS mice. Even if less robust than the difference in male NS and PRS social sniffing, the present reciprocal social interaction results are importantly the first report of atypical social behavior in female C57BL/6J PRS mice.

Although it is tempting to discuss the appearance of a potentially more robust overall social deficit in male versus female PRS mice in the context of sex differences in ASD prevalence, it remains unknown whether female PRS mice might engage in atypical social behavior in other contexts or forms not investigated in the current experiments (e.g., ultrasonic vocalizations). Therefore, the most salient conclusion to draw from the present investigation of social behavior in PRS mice is that the environmental risk factor of exposure to prenatal stress interestingly promoted the development of ASD-relevant social endophenotypes in both sexes of a mouse model for a clinical condition diagnosed four times more frequently in males than females.

In contrast to its effect on sociability, CLZ treatment did not rescue increased marble burying behavior in male PRS mice. The washout period instituted between the final CLZ injection and behavioral testing indicated that the treatment effects on behavior were unlikely due to residual psychoactive effects of the drug, but rather the consequence of CLZ-induced epigenetic modifications. RNA fluorescent barcoding findings supported the notion that sub-chronic CLZ treatment impacted epigenetic mechanisms. That CLZ treatment attenuated the male PRS sociability deficit but not the increased marble burying behavior suggested that the genes governing social and repetitive behaviors were regulated by different epigenetic mechanisms in C57BL/6J mice. It is possible that the PRS marble burying endophenotype was the product of an overexpression of its governing genes, thus rendering the epigenetic effects of CLZ behaviorally moot. The finding that CLZ increased marble burying behavior in NS vs. PRS mice was surprising and not readily interpretable. However, evidence linking specific epigenetic mechanisms to repetitive motor behaviors is scarce.

While CLZ is a broad-spectrum antipsychotic, existing literature reports that CLZ invokes epigenetic processes that function to facilitate a more open and transcriptionally permissive chromatin conformation, such as DNA demethylation, histone H3K9,14ac and H3K4me3 (Huang et al., 2007; Dong et al., 2008; Guidotti et al., 2011). The detection of increased *Mecp2* expression following CLZ treatment was here suggestive of a potential increase in DNA methylation and incongruent with prior linkage of CLZ to DNA demethylation (Dong et al., 2008; Matrisciano et al., 2011). However, prior work differs from the present study in dosage, vehicle, route of administration, genetic background of the mice investigated, and the interaction of that genetic background with stress. This observation of differing drug effects in what amounts to differing biological contexts support our premise that there exists a need for the study of the effects of PRS on the same genetic background used to generate most other mouse models for the study of ASD. At the same time, findings that were consistent with prior evidence of CLZ epigenetic mechanisms (Huang et al., 2007), such as the increased expression of *Kmt2c*, may be of particular interest for future investigations targeting the development of clinical therapeutics.

The present study of glutamate and GABA gene marker expression in the Hpc, Amg, NAc, mPFC, and CPu of male NS and PRS mice substantially expanded the current literature on molecular consequences of prenatal stress on E/I balance in ASD-relevant neurocircuitry with respect to the C57BL/6J genetic background. Previous work in this area has thus far been limited to measurement of the genes Grin2b and Kif17, respectively encoding the NR2B NMDA receptor subunit and its trafficking protein, in the Hpc (Zhao et al., 2013). The molecular investigation presented here indicated that the NAc and Amg might have been particularly vulnerable to the effects of prenatal stress. Of the mRNAs examined, there were significant reductions in the expression of excitatory-related genes in the Amg as compared with GABAergic-related genes. In the NAc, decreased expression of Grin2B and Gls are counterbalanced by increases in Gad2, Reln, and Pvalb. We also noted the lack of any changes in excitatory genes in the Hpc with increased expression of Reln and Gat1. Surprisingly, the only difference noted in the mPFC was an increase in the levels of Gad2 mRNA and no changes were noted in the CPu. Overall, it appears that there is a shift to an increased expression of inhibitory genes and a decrease in the expression of excitatory related mRNAs. However, it will be important in future studies to build upon this initial framework by both including a more comprehensive analysis and including female samples in all molecular analyses to allow a more direct examination of the functional and synaptic-level consequences of observed changes in glutamatergic and GABAergic marker genes.

The behavioral symptoms of ASD have long been hypothesized, broadly, to occur as the result of E/I imbalance in the brain (Hussman, 2001; Rubenstein and Merzenich, 2003). Identification of the direction of a shift in E/I balance in PRS mice was of particular interest to the present investigation due to the possibility that relationships between different etiological factors and E/I directionality might underlie the clinical heterogeneity of individuals with ASD. Furthermore, efforts to treat and develop treatments for ASD symptoms through modulation of glutamatergic and/or GABAergic systems would presumably benefit substantially from knowledge of such etiological factor–E/I directionality relationships. Clinical trials for pharmacotherapies designed to modulate E/I balance in individuals with ASD predominantly focus on reducing excess excitatory neurotransmission (e.g., memantine, gabapentin, AZD7325, lovastatin, STX209, N-acetylcysteine, riluzole). As such, the indication from the findings of the present study that inhibition may have exceeded excitation in the PRS mouse brain might provide guidance for improving treatment outcomes in individuals exposed to prenatal stress, whose symptoms might be unimproved or exacerbated following a glutamate receptor antagonist or GABA receptor agonist treatment.

In summary, we have characterized the male and female behavioral phenotypes of adult C57BL/6 mice exposed to gestational stress from prenatal days 8/9 through birth. Both male and female PRS mice exhibited less social sniffing in the reciprocal social interaction test, although only male PRS mice additionally exhibited reduced sociability in the three-chamber test of social interaction. Results of marble burying assays showed both male and female PRS increases in this RRB. No differences in anxiety-like behavior were observed in either male or female PRS mice. Interestingly, the antipsychotic CLZ that has been shown to have epigenetic-like properties reversed the sociability deficit but not the increased marble burying of male PRS mice. Changes in the expression of numerous genes related to glutamatergic and GABAergic neurons were measured in the Amg, NAc, Hpc, mPFC, and CPu. The observed changes, particularly in the Amg, are suggestive of a change in the balance of excitation/inhibition toward increased inhibitory activity.

## Synthesis

Reviewing Editor: Laura Volpicelli-Daley, The University of Alabama at Birmingham Decisions are customarily a result of the Reviewing Editor and the peer reviewers coming together and discussing their recommendations until a consensus is reached. When revisions are invited, a fact-based synthesis statement explaining their decision and outlining what is needed to prepare a revision will be listed below. The following reviewer(s) agreed to reveal their identity: Nour Al-Muhtasib. Note: If this manuscript was transferred from JNeurosci and a decision was made to accept the manuscript without peer review, a brief statement to this effect will instead be what is listed below.

**Overview**: As performed in other mouse strains and also in rats, the authors of this manuscript aimed to characterize autism spectrum disorder (ASD)-relevant behavioral and molecular phenotypes that follows prenatal stress in C5BL/6J mice. This study is of importance, as C5BL/6J mice are often used in preclinical studies that investigate the role of stress in many developmental neuropsychiatric disorders, including ASD. This study will likely advance the field by shedding light on potential sex-specific epigenetic mechanisms that may reduce ASD-like behaviors pending significant revision to the current manuscript.

**Major Concerns**

Abstract

Page 2 - The abstract requires significant reorganization and should be inclusive of the body or work presented in the manuscript. The behavioral tests and molecular assays should be highlighted.

Methods

Page 6 - In the second paragraph, please state exactly how many male and female mice were used. Additionally, please discuss how litter effects were taken into consideration.

Pages 7-9 -. Information related to time of day, order of testing, counterbalancing, etc. are missing completely. A timeline would increase comprehension. Some behavioral tests require greater detail (e.g., dimensions of the elevated plus maze).

Page 9 - Please operationally define grooming in the self-grooming assay.

Page 10 - If qRT-PCR experiments were only performed in male mice, please justify.

Page 11 - If the RNA Fluorescent Barcoding experiments were only performed in males, please justify.

Results

Pages 13-21 - Please provide the actual p-value and not just "p<0.01, etc).

Pages 13-21 - Please consider adding a sentence to each paragraph within the results section that explains the purpose of each experiment (similar to what was done for the figures)

Page 18 - Please explain why only males were assessed in the "Investigation of Epigenetic Alterations in PRS Mice through Behavioral Pharmacology".

Page 19 - Please present the RT-qPCR data in the order of Figure 11.

Pages 19-20 - Greater analysis of the gene expression data are required, as the table depicts a broader range of genes detected.

Discussion

Overall - It is important that the authors describe similarities or differences in gene expression patterns. Not all of the data are described in this way.

Page 23 - Please increase the discussion of why CLZ increased marble burying in NS mice.

Please discuss the findings in Figure 11.

Please add a summative paragraph to the discussion section.

Figures

All Figures - It is unclear if the N size is the same for each figure, please add this information to the figure captions or the figures themselves.

Figure 2 - Please label the graphs so that the sex of the mice is easily identified independent of the figure caption.

Tables

Page 55 - Please include all of the gene markers in Table 11-1 and identify (in bold) which makers are significantly different.

**Minor Concerns**

Methods

Page 7 - Please revise this the first sentence so that "CLZ" follows "A clozapine".

Results

Pages 13-21 - Please add a "0" before each decimal point. Many are missing.

Figures

Please be consistent in the presentation of the bar graphs. (+) and (-) error bars should be presented.

Tables

Page 47 - Table 1-1 should be revised to add greater context. Adding information related to the gene pool (e.g., glutamatergic or GABAergic gene markers) will increase readability.
