# Supplementary material for: Behavioral and Molecular Characterization of Prenatal Stress Effects on the C57BL/6J Genetic Background for the Study of Autism Spectrum Disorder
Source: eNeuro. 2024 Feb 9;11(2):ENEURO.0186-23.2024. doi: 10.1523/ENEURO.0186-23.2024 (PMC10897530; doi:10.1523/ENEURO.0186-23.2024)
Supplement: Table 1-1 — Full custom gene panel assessed for effects of prenatal stress exposure, CLZ treatment, and their interaction via RNA fluorescent barcoding (NanoString Technologies). Download Table 1-1, DOCX file. [file eneuro-11-ENEURO.0186-23.2024-s001.docx]

TABLE 1-1

| Custom RNA Fluorescent Barcoding Gene Panel | | | | | |
| --- | --- | --- | --- | --- | --- |
| DNA Methylation | DNA Demethylation | Histone Lysine Methylation | Histone Lysine Demethylation | PRS RNAseq-Derived | Neuroimmunity |
| *Dnmt1* | *Tet1* | *Smyd2* | *Kdm1a* | *Chat* | *Il17a* |
| *Dnmt3a* | *Tet2* | *Smyd3* | *Kdm1b* | *Drd1* | *Il1b* |
| *Dnmt3b* | *Tet3* | *Dot1l* | *Kdm2a* | *Drd2* | *Il4* |
| *Dnmt3l* | *Tdg* | *Nsd3* | *Kdm2b* | *Drd3* | *Il6* |
| *Mecp2* | *Gadd45b* | *Jarid2* | *Kdm3a* | *Drd4* | *Il10* |
| *Mbd2* |  | *Prdm8* | *Kdm3b* | *Drd5* | *Ifng* |
| *Mbd4* |  | *Prdm16* | *Kdm4a* | *Gdnf* | *Nfkb (Rela)* |
|  |  | *Ash1l* | *Kdm4b* | *iNOS* | *Tlr2* |
| Histone Acetylation | Histone Deacetylation | *Ash2l* | *Kdm4c* | *nNOS* | *Tlr4* |
| *Hat1* | *Hdac1* | *Mecom* | *Kdm4d* | *Pdyn* | *Tnfa* |
| *Kat6a* | *Hdac2* | *Kmt2a* | *Kdm5a* | *Penk* |  |
| *Kat6b* | *Hdac3* | *Kmt2b* | *Kdm5b* | *Rims1* | Sociability |
| *Kat7* | *Hdac4* | *Kmt2c* | *Kdm5c* | *Slc32a1* | *Avpr1a* |
| *Kat8* | *Hdac5* | *Kmt2d* | *Kdm5d* | *Sox9* | *Avpr1b* |
|  | *Hdac6* | *Suv39h1* | *Kdm6a* |  | *Avpr2* |
|  | *Hdac7* | *Suv39h2* | *Kdm6b* |  | *Oxtr* |
|  | *Hdac8* | *Setd1a* | *Kdm7a* |  |  |
|  | *Hdac9* | *Setd5* | *Phf2* |  | Stress Response |
|  | *Hdac10* | *Setd7* | *Phf8* |  | *Nr3c1* |
|  | *Hdac11* | *Setdb1* | *Jmjd1c* |  |  |
|  |  |  | *Hr* |  |  |
