# Supplementary material for: Behavioral and Molecular Characterization of Prenatal Stress Effects on the C57BL/6J Genetic Background for the Study of Autism Spectrum Disorder
Source: eNeuro. 2024 Feb 9;11(2):ENEURO.0186-23.2024. doi: 10.1523/ENEURO.0186-23.2024 (PMC10897530; doi:10.1523/ENEURO.0186-23.2024)
Supplement: Table 1-2 — Expanded RNA fluorescent barcoding results of interaction and main effects for prenatal stress exposure and CLZ treatment the in the NS and PRS NAc. Download Table 1-2, DOCX file. [file eneuro-11-ENEURO.0186-23.2024-s002.docx]

TABLE 1-2

| Gene | Effect | p-value | FDR |  |  |
| --- | --- | --- | --- | --- | --- |
| *Drd4* | Main Effect CLZ | 4.7846E-06 | 0.0008 |  | FDR < .05 |
| *Kmt2c* | Main Effect CLZ | 5.3504E-06 | 0.0008 |  | p < .01 |
| *Mecp2* | Main Effect CLZ | 8.1930E-06 | 0.0008 |  | p < .05 |
| *Nfkb (Rela)* | Main Effect CLZ | 3.5653E-05 | 0.0023 |  |  |
| *Phf2* | Main Effect CLZ | 4.3315E-05 | 0.0023 |  |  |
| *Hdac5* | Main Effect CLZ | 4.6193E-05 | 0.0023 |  |  |
| *Hdac6* | Main Effect CLZ | 0.0001 | 0.0058 |  |  |
| *Kat8* | Main Effect CLZ | 0.0002 | 0.0068 |  |  |
| *Setdb1* | Main Effect CLZ | 0.0002 | 0.0079 |  |  |
| *Smyd3* | Main Effect CLZ | 0.0005 | 0.0156 |  |  |
| *Kdm4a* | Main Effect CLZ | 0.0006 | 0.0159 |  |  |
| *Kmt2b* | Main Effect CLZ | 0.0018 | 0.0442 |  |  |
| *iNOS* | Main Effect CLZ | 0.0024 | 0.0510 |  |  |
| *Kdm5c* | Main Effect CLZ | 0.0024 | 0.0510 |  |  |
| *Setd1a* | Main Effect CLZ | 0.0045 | 0.0885 |  |  |
| *Ifng* | Main Effect CLZ | 0.0070 | 0.1256 |  |  |
| *Kmt2d* | Main Effect CLZ | 0.0073 | 0.1256 |  |  |
| *Setd7* | Main Effect Stress | 0.0091 | 0.1487 |  |  |
| *Hdac2* | Main Effect CLZ | 0.0102 | 0.1577 |  |  |
| *Hdac5* | Main Effect Stress | 0.0134 | 0.1971 |  |  |
| *Gadd45b* | Interaction | 0.0195 | 0.2734 |  |  |
| *Dnmt3a* | Main Effect CLZ | 0.0217 | 0.2772 |  |  |
| *Kdm2b* | Main Effect CLZ | 0.0217 | 0.2772 |  |  |
| *Il1b* | Main Effect CLZ | 0.0242 | 0.2887 |  |  |
| *Setd1a* | Main Effect Stress | 0.0254 | 0.2887 |  |  |
| *Kdm1a* | Main Effect Stress | 0.0255 | 0.2887 |  |  |
| *Il6* | Main Effect Stress | 0.0270 | 0.2926 |  |  |
| *Tlr4* | Main Effect CLZ | 0.0279 | 0.2926 |  |  |
| *Nfkb (Rela)* | Main Effect Stress | 0.0316 | 0.3205 |  |  |
| *Setd7* | Main Effect CLZ | 0.0342 | 0.3350 |  |  |
| *Ash2l* | Main Effect CLZ | 0.0357 | 0.3373 |  |  |
| *Smyd2* | Main Effect Stress | 0.0367 | 0.3373 |  |  |
| *Hdac6* | Main Effect Stress | 0.0402 | 0.3586 |  |  |
| *Tet1* | Main Effect CLZ | 0.0426 | 0.3673 |  |  |
| *Avpr2* | Main Effect Stress | 0.0437 | 0.3673 |  |  |
| *Kdm6b* | Main Effect Stress | 0.0530 | 0.4331 |  |  |
| *Kmt2c* | Interaction | 0.0583 | 0.4343 |  |  |
| *Kdm4b* | Main Effect CLZ | 0.0586 | 0.4343 |  |  |
| *Oxtr* | Main Effect Stress | 0.0592 | 0.4343 |  |  |
| *Hdac9* | Main Effect Stress | 0.0604 | 0.4343 |  |  |
| *Mbd4* | Main Effect CLZ | 0.0606 | 0.4343 |  |  |
| *Kdm6b* | Main Effect CLZ | 0.0654 | 0.4486 |  |  |
| *Tet3* | Main Effect CLZ | 0.0656 | 0.4486 |  |  |
| *Tet2* | Main Effect CLZ | 0.0710 | 0.4741 |  |  |
| *Kmt2d* | Main Effect Stress | 0.0741 | 0.4842 |  |  |
| *Tlr2* | Main Effect CLZ | 0.0791 | 0.5024 |  |  |
| *Il4* | Main Effect CLZ | 0.0803 | 0.5024 |  |  |
| *Ifng* | Interaction | 0.0839 | 0.5137 |  |  |
| *Il4* | Interaction | 0.0868 | 0.5137 |  |  |
| *Dnmt3l* | Main Effect CLZ | 0.0878 | 0.5137 |  |  |
| *Hdac7* | Main Effect CLZ | 0.0891 | 0.5137 |  |  |
| *Il10* | Main Effect CLZ | 0.0972 | 0.5384 |  |  |
| *Suv39h2* | Interaction | 0.0989 | 0.5384 |  |  |
| *Hdac8* | Main Effect CLZ | 0.0989 | 0.5384 |  |  |
| *Ash1l* | Main Effect CLZ | 0.1052 | 0.5556 |  |  |
| *Hdac7* | Main Effect Stress | 0.1072 | 0.5556 |  |  |
| *Kdm5c* | Interaction | 0.1077 | 0.5556 |  |  |
| *Tlr4* | Interaction | 0.1116 | 0.5570 |  |  |
| *Drd2* | Main Effect CLZ | 0.1133 | 0.5570 |  |  |
| *Setd5* | Main Effect CLZ | 0.1148 | 0.5570 |  |  |
| *Hdac10* | Main Effect CLZ | 0.1156 | 0.5570 |  |  |
| *Nfkb (Rela)* | Interaction | 0.1283 | 0.6085 |  |  |
| *Avpr1b* | Main Effect Stress | 0.1325 | 0.6177 |  |  |
| *Oxtr* | Main Effect CLZ | 0.1345 | 0.6177 |  |  |
| *Hr* | Main Effect Stress | 0.1369 | 0.6194 |  |  |
| *Drd4* | Main Effect Stress | 0.1408 | 0.6273 |  |  |
| *Il1b* | Main Effect Stress | 0.1486 | 0.6470 |  |  |
| *Il6* | Main Effect CLZ | 0.1497 | 0.6470 |  |  |
| *Hdac11* | Interaction | 0.1521 | 0.6479 |  |  |
| *Il17a* | Main Effect Stress | 0.1565 | 0.6481 |  |  |
| *Kmt2b* | Interaction | 0.1565 | 0.6481 |  |  |
| *Penk* | Main Effect Stress | 0.1679 | 0.6857 |  |  |
| *Ash2l* | Main Effect Stress | 0.1751 | 0.6901 |  |  |
| *Gdnf* | Interaction | 0.1774 | 0.6901 |  |  |
| *Kmt2c* | Main Effect Stress | 0.1779 | 0.6901 |  |  |
| *Kdm6b* | Interaction | 0.1784 | 0.6901 |  |  |
| *Kdm4c* | Main Effect Stress | 0.1839 | 0.7023 |  |  |
| *Hdac10* | Main Effect Stress | 0.1917 | 0.7205 |  |  |
| *Pdyn* | Main Effect Stress | 0.2013 | 0.7205 |  |  |
| *Nr3c1* | Interaction | 0.2048 | 0.7205 |  |  |
| *Avpr1a* | Interaction | 0.2069 | 0.7205 |  |  |
| *Dnmt1* | Main Effect Stress | 0.2077 | 0.7205 |  |  |
| *Rims1* | Main Effect CLZ | 0.2091 | 0.7205 |  |  |
| *Dot1l* | Interaction | 0.2102 | 0.7205 |  |  |
| *Nsd3* | Main Effect CLZ | 0.2140 | 0.7205 |  |  |
| *Phf8* | Main Effect Stress | 0.2154 | 0.7205 |  |  |
| *Dot1l* | Main Effect CLZ | 0.2158 | 0.7205 |  |  |
| *Mecom* | Main Effect Stress | 0.2174 | 0.7205 |  |  |
| *Jmjd1c* | Main Effect Stress | 0.2181 | 0.7205 |  |  |
| *Kdm2a* | Main Effect CLZ | 0.2274 | 0.7235 |  |  |
| *Jarid2* | Interaction | 0.2285 | 0.7235 |  |  |
| *Kdm6a* | Main Effect Stress | 0.2301 | 0.7235 |  |  |
| *Il17a* | Main Effect CLZ | 0.2318 | 0.7235 |  |  |
| *Rims1* | Main Effect Stress | 0.2334 | 0.7235 |  |  |
| *Hdac7* | Interaction | 0.2385 | 0.7235 |  |  |
| *Kdm2a* | Interaction | 0.2399 | 0.7235 |  |  |
| *Jarid2* | Main Effect CLZ | 0.2412 | 0.7235 |  |  |
| *Smyd2* | Main Effect CLZ | 0.2439 | 0.7235 |  |  |
| *Drd1* | Main Effect Stress | 0.2453 | 0.7235 |  |  |
| *Kdm5a* | Main Effect Stress | 0.2497 | 0.7235 |  |  |
| *Kat8* | Main Effect Stress | 0.2504 | 0.7235 |  |  |
| *Sox9* | Main Effect Stress | 0.2535 | 0.7235 |  |  |
| *Kat8* | Interaction | 0.2552 | 0.7235 |  |  |
| *Kat6a* | Interaction | 0.2559 | 0.7235 |  |  |
| *Phf8* | Main Effect CLZ | 0.2589 | 0.7251 |  |  |
| *Hdac4* | Interaction | 0.2671 | 0.7408 |  |  |
| *Mecom* | Interaction | 0.2705 | 0.7427 |  |  |
| *Kmt2b* | Main Effect Stress | 0.2728 | 0.7427 |  |  |
| *Hdac9* | Interaction | 0.2766 | 0.7461 |  |  |
| *Prdm8* | Main Effect Stress | 0.2830 | 0.7529 |  |  |
| *Hdac3* | Main Effect Stress | 0.2843 | 0.7529 |  |  |
| *Tlr4* | Main Effect Stress | 0.2915 | 0.7623 |  |  |
| *Kdm4b* | Interaction | 0.2935 | 0.7623 |  |  |
| *Drd2* | Interaction | 0.2956 | 0.7623 |  |  |
| *Mbd4* | Main Effect Stress | 0.2984 | 0.7630 |  |  |
| *Drd1* | Interaction | 0.3060 | 0.7710 |  |  |
| *Kdm4c* | Main Effect CLZ | 0.3080 | 0.7710 |  |  |
| *Kdm7a* | Interaction | 0.3150 | 0.7710 |  |  |
| *Tet1* | Main Effect Stress | 0.3191 | 0.7710 |  |  |
| *Dnmt3b* | Main Effect CLZ | 0.3197 | 0.7710 |  |  |
| *Hdac6* | Interaction | 0.3233 | 0.7710 |  |  |
| *Drd5* | Interaction | 0.3240 | 0.7710 |  |  |
| *Drd2* | Main Effect Stress | 0.3260 | 0.7710 |  |  |
| *Kmt2a* | Main Effect Stress | 0.3268 | 0.7710 |  |  |
| *Nr3c1* | Main Effect CLZ | 0.3278 | 0.7710 |  |  |
| *Suv39h2* | Main Effect CLZ | 0.3320 | 0.7748 |  |  |
| *Kdm4d* | Main Effect Stress | 0.3441 | 0.7825 |  |  |
| *Drd3* | Main Effect Stress | 0.3446 | 0.7825 |  |  |
| *Smyd3* | Interaction | 0.3521 | 0.7825 |  |  |
| *Kdm3a* | Main Effect Stress | 0.3525 | 0.7825 |  |  |
| *Setd5* | Interaction | 0.3550 | 0.7825 |  |  |
| *Avpr1b* | Main Effect CLZ | 0.3553 | 0.7825 |  |  |
| *Mecp2* | Main Effect Stress | 0.3600 | 0.7825 |  |  |
| *Avpr1a* | Main Effect CLZ | 0.3605 | 0.7825 |  |  |
| *Tet3* | Interaction | 0.3612 | 0.7825 |  |  |
| *Ash1l* | Main Effect Stress | 0.3620 | 0.7825 |  |  |
| *Kmt2d* | Interaction | 0.3698 | 0.7936 |  |  |
| *Mecom* | Main Effect CLZ | 0.3817 | 0.8027 |  |  |
| *Kdm4d* | Interaction | 0.3839 | 0.8027 |  |  |
| *Hdac4* | Main Effect CLZ | 0.3859 | 0.8027 |  |  |
| *Prdm8* | Main Effect CLZ | 0.3885 | 0.8027 |  |  |
| *Prdm16* | Main Effect CLZ | 0.3984 | 0.8027 |  |  |
| *Kdm7a* | Main Effect CLZ | 0.4042 | 0.8027 |  |  |
| *Penk* | Interaction | 0.4047 | 0.8027 |  |  |
| *Nr3c1* | Main Effect Stress | 0.4057 | 0.8027 |  |  |
| *Kdm6a* | Interaction | 0.4063 | 0.8027 |  |  |
| *Rims1* | Interaction | 0.4068 | 0.8027 |  |  |
| *Prdm16* | Main Effect Stress | 0.4103 | 0.8027 |  |  |
| *Ash2l* | Interaction | 0.4109 | 0.8027 |  |  |
| *Suv39h2* | Main Effect Stress | 0.4109 | 0.8027 |  |  |
| *Tdg* | Main Effect Stress | 0.4123 | 0.8027 |  |  |
| *iNOS* | Interaction | 0.4229 | 0.8180 |  |  |
| *Gdnf* | Main Effect Stress | 0.4296 | 0.8255 |  |  |
| *Chat* | Interaction | 0.4370 | 0.8272 |  |  |
| *Kdm1b* | Main Effect CLZ | 0.4371 | 0.8272 |  |  |
| *Kat7* | Interaction | 0.4391 | 0.8272 |  |  |
| *Tlr2* | Interaction | 0.4417 | 0.8272 |  |  |
| *Mbd2* | Interaction | 0.4492 | 0.8326 |  |  |
| *Kdm4b* | Main Effect Stress | 0.4503 | 0.8326 |  |  |
| *nNOS* | Main Effect Stress | 0.4573 | 0.8402 |  |  |
| *Drd5* | Main Effect Stress | 0.4755 | 0.8582 |  |  |
| *Kat6b* | Main Effect Stress | 0.4780 | 0.8582 |  |  |
| *Pdyn* | Interaction | 0.4781 | 0.8582 |  |  |
| *Il6* | Interaction | 0.4787 | 0.8582 |  |  |
| *Setdb1* | Interaction | 0.4840 | 0.8600 |  |  |
| *Dnmt3a* | Interaction | 0.4889 | 0.8600 |  |  |
| *Hat1* | Main Effect CLZ | 0.4914 | 0.8600 |  |  |
| *Suv39h1* | Main Effect Stress | 0.4914 | 0.8600 |  |  |
| *Tet3* | Main Effect Stress | 0.4946 | 0.8604 |  |  |
| *Dnmt3b* | Interaction | 0.5003 | 0.8619 |  |  |
| *Mecp2* | Interaction | 0.5013 | 0.8619 |  |  |
| *Mbd4* | Interaction | 0.5264 | 0.8749 |  |  |
| *Kdm3b* | Interaction | 0.5286 | 0.8749 |  |  |
| *Sox9* | Main Effect CLZ | 0.5297 | 0.8749 |  |  |
| *Dnmt1* | Main Effect CLZ | 0.5309 | 0.8749 |  |  |
| *Hdac1* | Main Effect CLZ | 0.5332 | 0.8749 |  |  |
| *Pdyn* | Main Effect CLZ | 0.5341 | 0.8749 |  |  |
| *Tnfa* | Main Effect Stress | 0.5354 | 0.8749 |  |  |
| *Dnmt3l* | Main Effect Stress | 0.5391 | 0.8749 |  |  |
| *Drd1* | Main Effect CLZ | 0.5402 | 0.8749 |  |  |
| *Kdm5b* | Interaction | 0.5423 | 0.8749 |  |  |
| *Kdm5b* | Main Effect CLZ | 0.5431 | 0.8749 |  |  |
| *Hdac8* | Interaction | 0.5478 | 0.8749 |  |  |
| *Tnfa* | Interaction | 0.5497 | 0.8749 |  |  |
| *Mbd2* | Main Effect Stress | 0.5510 | 0.8749 |  |  |
| *Nsd3* | Interaction | 0.5535 | 0.8749 |  |  |
| *Kat6a* | Main Effect Stress | 0.5574 | 0.8750 |  |  |
| *Hdac4* | Main Effect Stress | 0.5595 | 0.8750 |  |  |
| *Hdac10* | Interaction | 0.5636 | 0.8767 |  |  |
| *Suv39h* | Interaction | 0.5740 | 0.8883 |  |  |
| *Prdm16* | Interaction | 0.5798 | 0.8924 |  |  |
| *Hr* | Main Effect CLZ | 0.5890 | 0.9020 |  |  |
| *Tet2* | Interaction | 0.5951 | 0.9065 |  |  |
| *Smyd2* | Interaction | 0.6023 | 0.9075 |  |  |
| *Avpr2* | Main Effect CLZ | 0.6035 | 0.9075 |  |  |
| *Tdg* | Interaction | 0.6064 | 0.9075 |  |  |
| *Hat1* | Interaction | 0.6081 | 0.9075 |  |  |
| *Kdm5d* | Main Effect Stress | 0.6140 | 0.9117 |  |  |
| *Kdm3b* | Main Effect CLZ | 0.6196 | 0.9142 |  |  |
| *Kdm5a* | Interaction | 0.6219 | 0.9142 |  |  |
| *Tnfa* | Main Effect CLZ | 0.6291 | 0.9202 |  |  |
| *Penk* | Main Effect CLZ | 0.6341 | 0.9229 |  |  |
| *Kdm2a* | Main Effect Stress | 0.6418 | 0.9270 |  |  |
| *Phf8* | Interaction | 0.6435 | 0.9270 |  |  |
| *Dnmt3l* | Interaction | 0.6467 | 0.9270 |  |  |
| *Hr* | Interaction | 0.6496 | 0.9270 |  |  |
| *Dot1l* | Main Effect Stress | 0.6553 | 0.9307 |  |  |
| *Hdac1* | Interaction | 0.6612 | 0.9345 |  |  |
| *Tlr2* | Main Effect Stress | 0.6757 | 0.9505 |  |  |
| *Hat1* | Main Effect Stress | 0.6883 | 0.9596 |  |  |
| *Mbd2* | Main Effect CLZ | 0.6887 | 0.9596 |  |  |
| *Tdg* | Main Effect CLZ | 0.6978 | 0.9658 |  |  |
| *Kat6a* | Main Effect CLZ | 0.6997 | 0.9658 |  |  |
| *Il4* | Main Effect Stress | 0.7075 | 0.9720 |  |  |
| *Kdm4c* | Interaction | 0.7142 | 0.9766 |  |  |
| *Kdm2b* | Main Effect Stress | 0.7251 | 0.9828 |  |  |
| *Slc32a1* | Interaction | 0.7313 | 0.9828 |  |  |
| *Kdm5b* | Main Effect Stress | 0.7326 | 0.9828 |  |  |
| *Kdm3b* | Main Effect Stress | 0.7365 | 0.9828 |  |  |
| *Kdm3a* | Main Effect CLZ | 0.7384 | 0.9828 |  |  |
| *Dnmt3b* | Main Effect Stress | 0.7407 | 0.9828 |  |  |
| *Ifng* | Main Effect Stress | 0.7485 | 0.9828 |  |  |
| *nNOS* | Interaction | 0.7502 | 0.9828 |  |  |
| *nNOS* | Main Effect CLZ | 0.7504 | 0.9828 |  |  |
| *Kat7* | Main Effect Stress | 0.7521 | 0.9828 |  |  |
| *Tet2* | Main Effect Stress | 0.7607 | 0.9860 |  |  |
| *Kmt2a* | Interaction | 0.7647 | 0.9860 |  |  |
| *Drd4* | Interaction | 0.7668 | 0.9860 |  |  |
| *Kat7* | Main Effect CLZ | 0.7680 | 0.9860 |  |  |
| *Tet1* | Interaction | 0.7719 | 0.9867 |  |  |
| *Avpr1b* | Interaction | 0.7791 | 0.9895 |  |  |
| *Kdm1a* | Main Effect CLZ | 0.7809 | 0.9895 |  |  |
| *Kdm5a* | Main Effect CLZ | 0.7875 | 0.9937 |  |  |
| *Kdm5c* | Main Effect Stress | 0.7934 | 0.9939 |  |  |
| *Slc32a1* | Main Effect CLZ | 0.7944 | 0.9939 |  |  |
| *Gadd45b* | Main Effect CLZ | 0.8001 | 0.9952 |  |  |
| *Hdac9* | Main Effect CLZ | 0.8022 | 0.9952 |  |  |
| *Drd3* | Main Effect CLZ | 0.8101 | 0.9971 |  |  |
| *iNOS* | Main Effect Stress | 0.8167 | 0.9971 |  |  |
| *Avpr1a* | Main Effect Stress | 0.8241 | 0.9971 |  |  |
| *Hdac11* | Main Effect CLZ | 0.8412 | 0.9971 |  |  |
| *Kdm5d* | Interaction | 0.8443 | 0.9971 |  |  |
| *Hdac5* | Interaction | 0.8532 | 0.9971 |  |  |
| *Gdnf* | Main Effect CLZ | 0.8536 | 0.9971 |  |  |
| *Ash1l* | Interaction | 0.8556 | 0.9971 |  |  |
| *Hdac8* | Main Effect Stress | 0.8574 | 0.9971 |  |  |
| *Kat6b* | Interaction | 0.8609 | 0.9971 |  |  |
| *Kdm6a* | Main Effect CLZ | 0.8629 | 0.9971 |  |  |
| *Kdm1a* | Interaction | 0.8634 | 0.9971 |  |  |
| *Kdm4d* | Main Effect CLZ | 0.8668 | 0.9971 |  |  |
| *Kmt2a* | Main Effect CLZ | 0.8688 | 0.9971 |  |  |
| *Hdac1* | Main Effect Stress | 0.8791 | 0.9971 |  |  |
| *Hdac2* | Interaction | 0.8821 | 0.9971 |  |  |
| *Setdb1* | Main Effect Stress | 0.8828 | 0.9971 |  |  |
| *Nsd3* | Main Effect Stress | 0.8847 | 0.9971 |  |  |
| *Kdm2b* | Interaction | 0.8865 | 0.9971 |  |  |
| *Kdm4a* | Interaction | 0.8914 | 0.9971 |  |  |
| *Gadd45b* | Main Effect Stress | 0.8924 | 0.9971 |  |  |
| *Phf2* | Main Effect Stress | 0.8925 | 0.9971 |  |  |
| *Setd7* | Interaction | 0.8931 | 0.9971 |  |  |
| *Suv39h1* | Main Effect CLZ | 0.8944 | 0.9971 |  |  |
| *Kdm1b* | Main Effect Stress | 0.8947 | 0.9971 |  |  |
| *Jarid2* | Main Effect Stress | 0.8982 | 0.9971 |  |  |
| *Chat* | Main Effect CLZ | 0.8993 | 0.9971 |  |  |
| *Avpr2* | Interaction | 0.9054 | 0.9971 |  |  |
| *Kdm5d* | Main Effect CLZ | 0.9061 | 0.9971 |  |  |
| *Dnmt1* | Interaction | 0.9100 | 0.9971 |  |  |
| *Hdac3* | Main Effect CLZ | 0.9122 | 0.9971 |  |  |
| *Il1b* | Interaction | 0.9202 | 0.9971 |  |  |
| *Dnmt3a* | Main Effect Stress | 0.9249 | 0.9971 |  |  |
| *Chat* | Main Effect Stress | 0.9272 | 0.9971 |  |  |
| *Drd5* | Main Effect CLZ | 0.9300 | 0.9971 |  |  |
| *Phf2* | Interaction | 0.9306 | 0.9971 |  |  |
| *Kdm1b* | Interaction | 0.9352 | 0.9971 |  |  |
| *Il10* | Main Effect Stress | 0.9379 | 0.9971 |  |  |
| *Prdm8* | Interaction | 0.9432 | 0.9971 |  |  |
| *Setd5* | Main Effect Stress | 0.9476 | 0.9971 |  |  |
| *Hdac2* | Main Effect Stress | 0.9526 | 0.9971 |  |  |
| *Drd3* | Interaction | 0.9534 | 0.9971 |  |  |
| *Sox9* | Interaction | 0.9583 | 0.9971 |  |  |
| *Hdac11* | Main Effect Stress | 0.9592 | 0.9971 |  |  |
| *Slc32a1* | Main Effect Stress | 0.9644 | 0.9971 |  |  |
| *Kdm4a* | Main Effect Stress | 0.9659 | 0.9971 |  |  |
| *Jmjd1c* | Main Effect CLZ | 0.9670 | 0.9971 |  |  |
| *Oxtr* | Interaction | 0.9677 | 0.9971 |  |  |
| *Jmjd1c* | Interaction | 0.9711 | 0.9971 |  |  |
| *Smyd3* | Main Effect Stress | 0.9775 | 0.9971 |  |  |
| *Il17a* | Interaction | 0.9803 | 0.9971 |  |  |
| *Hdac3* | Interaction | 0.9832 | 0.9971 |  |  |
| *Kdm7a* | Main Effect Stress | 0.9838 | 0.9971 |  |  |
| *Kdm3a* | Interaction | 0.9869 | 0.9971 |  |  |
| *Il10* | Interaction | 0.9914 | 0.9971 |  |  |
| *Setd1a* | Interaction | 0.9937 | 0.9971 |  |  |
| *Kat6b* | Main Effect CLZ | 0.9971 | 0.9971 |  |  |
