# Supplementary material for: Behavioral and Molecular Characterization of Prenatal Stress Effects on the C57BL/6J Genetic Background for the Study of Autism Spectrum Disorder
Source: eNeuro. 2024 Feb 9;11(2):ENEURO.0186-23.2024. doi: 10.1523/ENEURO.0186-23.2024 (PMC10897530; doi:10.1523/ENEURO.0186-23.2024)
Supplement: Table 11-1 — Results of nonsignificant differences in mRNA expression levels of glutamatergic and GABAergic gene markers in the Hpc, NAc, Amg, mPFC, and CPu of NS and PRS mice presented in Figure 11. Download Table 11-1, DOCX file. [file eneuro-11-ENEURO.0186-23.2024-s003.docx]

| Brain Region | Marker Type | Gene | NS Mean FC (SEM) | PRS Mean FC (SEM) | p-value |
| --- | --- | --- | --- | --- | --- |
| Amygdala | Glutamatergic | *Vglut1* | 1.00 (0.12) | 0.86 (0.04) | 0.157 |
|  |  | *Vglut2* | 1.00 (0.10) | 0.84 (0.08) | 0.287 |
|  |  | ***Grin1*** | **1.00 (0.06)** | **0.65 (0.05)** | **0.001** |
|  |  | ***Grin2b*** | **1.00 (0.06)** | **0.61 (0.07)** | **0.002** |
|  |  | ***Gls*** | **1.00 (0.04)** | **0.65 (0.05)** | **0.0001** |
|  |  | ***Gls2*** | **1.00 (0.07)** | **0.64 (0.06)** | **0.003** |
|  |  | ***Glul*** | **1.00 (0.07)** | **0.69 (0.04)** | **0.010** |
|  | GABAergic | *Gad1* | 1.00 (0.11) | 0.72 (0.08) | 0.080 |
|  |  | *Gad2* | 1.00 (0.09) | 0.94 (0.16) | 0.712 |
|  |  | *Reln* | 1.00 (0.06) | 0.92 (0.14) | 0.599 |
|  |  | *Pvalb* | 1.00 (0.12) | 0.72 (0.12) | 0.147 |
|  |  | *Sst* | 1.00 (0.09) | 1.14 (0.09) | 0.299 |
|  |  | ***Gat1*** | **1.00 (0.11)** | **0.60 (0.07)** | **0.018** |
|  |  | *Gat3* | 1.00 (0.06) | 0.82 (0.08) | 0.084 |
| Nucleus Accumbens | Glutamatergic | *Vglut1* | 1.00 (0.29) | 1.39 (0.27) | 0.359 |
|  |  | *Vglut2* | 1.00 (0.20) | 0.70 (0.19) | 0.317 |
|  |  | *Grin1* | 1.00 (0.05) | 1.05 (0.02) | 0.400 |
|  |  | ***Grin2b*** | **1.00 (0.18)** | **0.54 (0.06)** | **0.047** |
|  |  | ***Gls*** | **1.00 (0.08)** | **0.74 (0.03)** | **0.029** |
|  |  | *Gls2* | 1.00 (0.06) | 1.04 (0.15) | 0.761 |
|  |  | *Glul* | 1.00 (0.07) | 0.97 (0.14) | 0.846 |
|  | GABAergic | *Gad1* | 1.00 (0.05) | 1.05 (0.04) | 0.537 |
|  |  | ***Gad2*** | **1.00 (0.05)** | **1.16 (0.02)** | **0.025** |
|  |  | ***Reln*** | **1.00 (0.14)** | **1.57 (0.12)** | **0.015** |
|  |  | ***Pvalb*** | **1.00 (0.06)** | **1.31 (0.10)** | **0.024** |
|  |  | *Sst* | 1.00 (0.10) | 0.96 (0.09) | 0.775 |
|  |  | *Gat1* | 1.00 (0.06) | 1.05 (0.06) | 0.527 |
|  |  | *Gat3* | 1.00 (0.09) | 0.99 (0.11) | 0.921 |
| Hippocampus | Glutamatergic | *Vglut1* | 1.00 (0.05) | 1.15 (0.06) | 0.072 |
|  |  | *Vglut2* | 1.00 (0.19) | 0.75 (0.22) | 0.416 |
|  |  | *Grin1* | 1.00 (0.04) | 1.03 (0.06) | 0.681 |
|  |  | *Grin2b* | 1.00 (0.06) | 1.07 (0.06) | 0.433 |
|  |  | *Gls* | 1.00 (0.03) | 0.95 (0.07) | 0.420 |
|  |  | *Gls2* | 1.00 (0.04) | 0.94 (0.08) | 0.470 |
|  |  | *Glul* | 1.00 (0.05) | 0.98 (0.04) | 0.711 |
|  | GABAergic | *Gad1* | 1.00 (0.03) | 1.16 (0.08) | 0.068 |
|  |  | *Gad2* | 1.00 (0.05) | 1.34 (0.15) | 0.082 |
|  |  | ***Reln*** | **1.00 (0.07)** | **1.33 (0.13)** | **0.035** |
|  |  | *Pvalb* | 1.00 (0.08) | 0.95 (0.12) | 0.713 |
|  |  | *Sst* | 1.00 (0.10) | 1.37 (0.19) | 0.082 |
|  |  | ***Gat1*** | **1.00 (0.03)** | **1.28 (0.03)** | **0.0005** |
|  |  | *Gat3* | 1.00 (0.09) | 1.05 (0.09) | 0.718 |
| Medial Prefrontal Cortex | Glutamatergic | *Vglut1* | 1.00 (0.07) | 0.89 (0.04) | 0.254 |
|  |  | *Vglut2* | 1.00 (0.12) | 0.69 (0.07) | 0.065 |
|  |  | *Grin1* | 1.00 (0.07) | 0.94 (0.07) | 0.538 |
|  |  | *Grin2b* | 1.00 (0.09) | 1.25 (0.18) | 0.213 |
|  |  | *Gls* | 1.00 (0.04) | 0.92 (0.03) | 0.191 |
|  |  | *Gls2* | 1.00 (0.04) | 1.08 (0.06) | 0.283 |
|  |  | *Glul* | 1.00 (0.04) | 0.91 (0.06) | 0.191 |
|  | GABAergic | *Gad1* | 1.00 (0.08) | 1.02 (0.06) | 0.889 |
|  |  | ***Gad2*** | **1.00 (0.06)** | **1.36 (0.11)** | **0.012** |
|  |  | *Reln* | 1.00 (0.12) | 0.79 (0.05) | 0.188 |
|  |  | *Pvalb* | 1.00 (0.07) | 1.02 (0.08) | 0.829 |
|  |  | *Sst* | 1.00 (0.06) | 1.06 (0.06) | 0.500 |
|  |  | *Gat1* | 1.00 (0.07) | 0.89 (0.06) | 0.273 |
|  |  | *Gat3* | 1.00 (0.06) | 0.96 (0.06) | 0.684 |
| Caudate-Putamen | Glutamatergic | *Vglut1* | 1.00 (0.20) | 0.96 (0.34) | 0.920 |
|  |  | *Vglut2* | 1.00 (0.25) | 1.23 (0.31) | 0.581 |
|  |  | *Grin1* | 1.00 (0.03) | 0.99 (0.03) | 0.877 |
|  |  | *Grin2b* | 1.00 (0.04) | 0.92 (0.05) | 0.249 |
|  |  | *Gls* | 1.00 (0.08) | 0.89 (0.06) | 0.338 |
|  |  | *Gls2* | 1.00 (0.06) | 1.06 (0.08) | 0.534 |
|  |  | *Glul* | 1.00 (0.04) | 1.06 (0.02) | 0.251 |
|  | GABAergic | *Gad1* | 1.00 (0.05) | 1.11 (0.09) | 0.287 |
|  |  | *Gad2* | 1.00 (0.09) | 1.27 (0.11) | 0.080 |
|  |  | *Reln* | 1.00 (0.10) | 1.21 (0.18) | 0.285 |
|  |  | *Pvalb* | 1.00 (0.19) | 1.09 (0.14) | 0.724 |
|  |  | *Sst* | 1.00 (0.07) | 1.00 (0.13) | 0.989 |
|  |  | *Gat1* | 1.00 (0.05) | 0.94 (0.04) | 0.356 |
|  |  | *Gat3* | 1.00 (0.16) | 1.27 (0.20) | 0.313 |
